# Supplementary material for: Gene Functional Networks from Time Expression Profiles: A Constructive Approach Demonstrated in Chili Pepper (Capsicum annuum L.)
Source: Plants (Basel). 2023 Mar 3;12(5):1148. doi: 10.3390/plants12051148 (PMC10005043; doi:10.3390/plants12051148)
Supplement: Supplementary file 1 [file plants-12-01148-s001.zip › plants-2222623-supplementary.pdf]

SUPPLEMENTARY INFORMATION FOR "Gene Functional Networks from Time Expression Profiles: A constructive approach demonstrated in chili pepper"

ALAN FLORES-DÍAZ, CHRISTIAN ESCOTO-SANDOVAL, FELIPE CERVANTES-HERNÁNDEZ, JOSÉ J. ORDAZ-ORTIZ, CORINA HAYANO-KANASHIRO, HUMBERTO REYES-VALDÉS, ANA GARCÉS-CLAVER, NEFTALÍ OCHOA-ALEJO, AND OCTAVIO MARTÍNEZ

Note: Sections of this document are in the main text as "**SI.#**", where "#" is the number of the section or subsection in this document (Table of Contents below). Some of the figures that appear in the main text are also presented here.

Content

SI.1. Introduction: a "*hairy ball*" example 2
SI.2. Methods 2
SI.2.1. Congruent gene correlations 2
SI.2.2. Robustness of a relation between genes 4
SI.2.3. The "*Gene2Gene*" algorithm 6
SI.2.4. A general constructive approach 9
SI.2.5. Linking GFN modules 9
SI.3. Data analyses 10
SI.3.1. GFN for three biological processes (BPs) 10
SI.3.2. Sensitivity of the *Gene2Gene* algorithm to input parameters 13
SI.3.3. A "*Meta Network*" (MN) linking the GFN `rep`, `celcy` and `vir` 15
SI.3.4. Distinctive time expression profiles within the MN 18
SI.3.5. Finding Transcription Factors (TF) consistent with expression pattern **[P=2]** 25
SI.4. Concluding remarks 27
SI.4.1. Generality and limitations of the *Gene2Gene* algorithm 27
SI.4.2. Constructing GFN: a bottom-up approach 28
SI.5. Appendices 29
SI.5.1. R code 30
References 38

(AF-D, CE-S. FC-H, JJO-O and OM) Unidad de Genómica Avanzada (Langebio), Centro de Investigación y de Estudios Avanzados del Instituto Politécnico Nacional (Cinvestav), Irapuato Guanajuato, 36824, México.

(CH-K) Departamento de Investigaciones Científicas y Tecnológicas de la Universidad de Sonora, Hermosillo Sonora, 83000, México.

(HR-V) Department of Plant Breeding, Universidad Autónoma Agraria Antonio Narro, Saltillo Coahuila, 25315, México.

(AG-C) Unidad de Hortofruticultura, Centro de Investigación y Tecnología Agroalimentaria de Aragón. Instituto Agroalimentario de Aragón-IA2 (CITA-Universidad de Zaragoza), Zaragoza, 50059, Spain.

(NO-A) Departamento de Ingeniería Genética, Centro de Investigación y de Estudios Avanzados del Instituto Politécnico Nacional (Cinvestav), Irapuato Guanajuato, 36824, México.

E-mail addresses: `octavio.martinez@cinvestav.mx`.

Date: January 31, 2023.

SI.1. Introduction: a "*hairy ball*" example

Figure 1 presents an example of a plot of a large gene co-expression network (GCN) which illustrates the "*hairy ball*" effect.

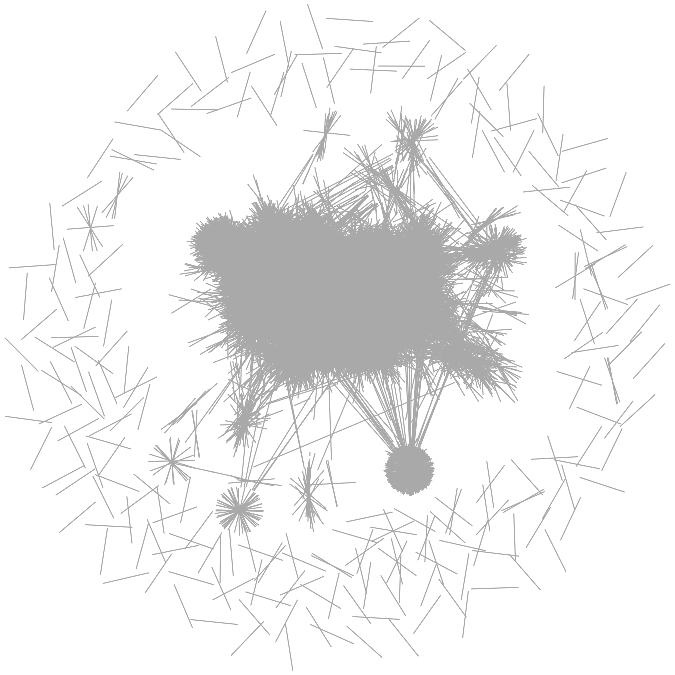

Figure 1. Example of a "*hairy ball*". Plot of a gene co-expression network containing 13193 genes and 38830 edges. Only edges are shown as grey lines.

Figure 1 was obtained by running ARACNE (Margolin et al., 2006) with 168 RNA-Seq libraries from chili pepper developing fruits in 12 different genotypes, each one of them evaluated at 7 times of fruit development and with 2 biological replicates per condition ($12 \times 7 \times 2 = 168$). Data are available in the R package "*Salsa*" (Martínez and Escoto-Sandoval, 2021), and are the same data used in the main text to demonstrate the *Gene2Gene* algorithm.

Given that this network contains 13193 genes and 38830 edges, its direct interpretation is impossible without simplification or zooming into a particular section of the network. In contrast, the *Gene2Gene* algorithm allows the optimum use of the data to obtain Gene Functional Networks (GFN) with a given level of robustness.

SI.2. Methods

SI.2.1. **Congruent gene correlations.** Given that the algorithm that we present here depends on SEP, we begin by giving an illustrative example of how and why the correlation between these summaries of significant expression changes are useful to detect relevant relations between genes.

A SEP is a numeric vector that summarizes all relevant statistical information about changes of expression within a sampled time interval Martinez (2022). To estimate the SEP of a gene from a given data source, we begin by performing tests of expression changes between neighbor intervals. Such tests take all replicates available to decide if, from one time point to the next, there is enough evidence of an expression change, or the gene is in a steady state "S". If there is evidence of change, then it could be only in two directions, increase, "I", or decrease, "D". The procedure ends by standardizing the

expression in all intervals to have a mean of 0 and a standard deviation of 1. A detailed description of this procedure, which is applicable to either RNA-Seq or microarray data, is given in Escoto-Sandoval et al. (2021a); Martinez (2022).

By been standardized, SEPs allow a fair pair-wise comparison of genes which could have very different expression levels, as for example genes coding for Transcription Factors (TF) –which usually have relative low expression, and genes coding for enzymes or other proteins –which could have relative expression orders of magnitude higher than TF. Figure 2 (Fig. 1 in main text) presents the plot of an example of SEPs for two genes that present highly correlated standardized expression in two different chili pepper genotypes, using data and functions available in the R package "*Salsa*" (Martínez and Escoto-Sandoval, 2021).

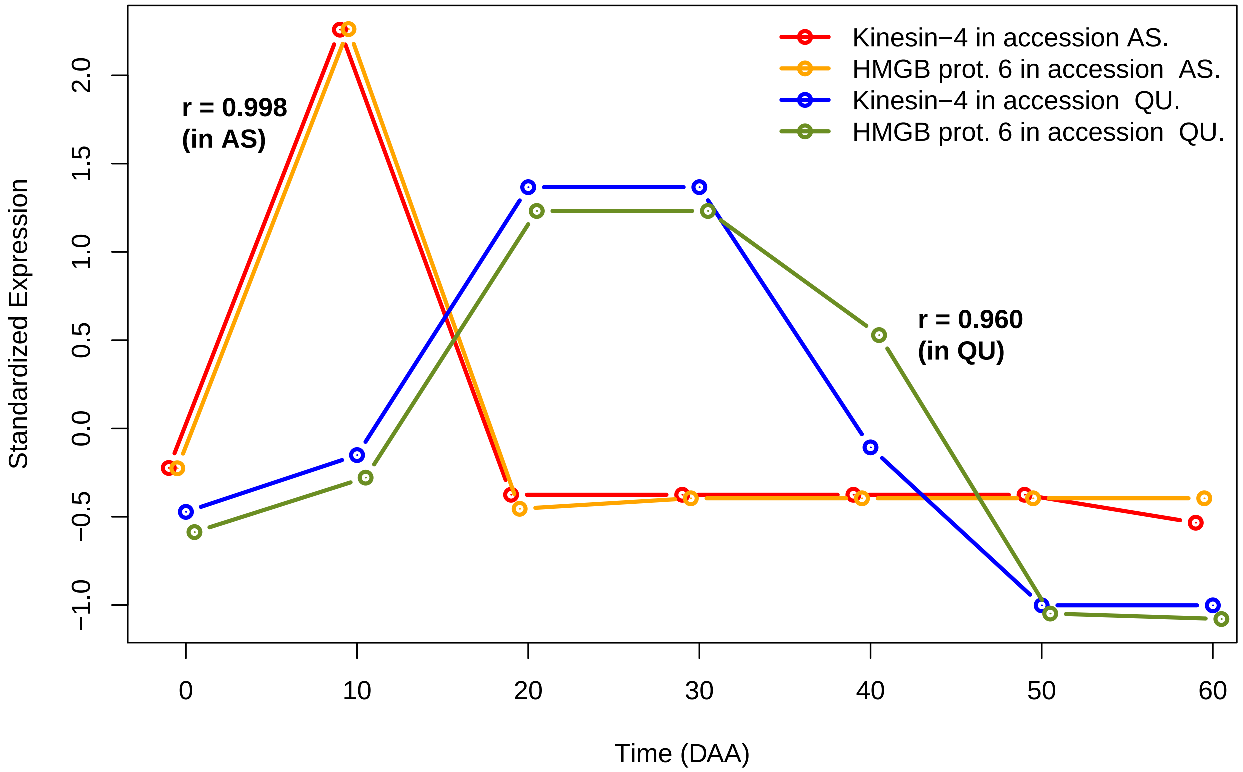

FIGURE 2. Example of SEPs for two genes highly correlated in two independent genotypes (Fig. 1 in main text).

Figure 2 shows SEPs for the genes coding for the motor protein "*kinesin-4*" with NCBI identifier XP_016552070.1 and for the TF "*High Mobility Group B protein 6*" with NCBI identifier XP_016555757.1, and abbreviated in the plot legend as "HMGB prot. 6". The X-axis of the plot show time, given in Days After Anthesis (DAA), while the Y-axis gives the standardized gene expression. SEPs are presented for chili pepper genotypes "AS" (Ancho San Luis) and "QU" (Querétaro), which correspond to a domesticated and wild chili pepper genotypes, respectively.

In Figure 2 we can appreciate how the expression profiles of the two genes are highly concordant when compared within the same chili pepper genotype, but highly divergent in the two genotypes. The SEPs for the kinesin-4 and HMGB prot. 6 in genotype AS (red and orange lines) are almost equal, having both a global maximum at 10 DAA, decreasing sharply from 10 to 20 DAA and then staying in an almost steady state until the end of the time period sampled, at 60 DAA. In contrast, the SEPs for those two genes in the genotype QU (blue and green lines) increase from 0 up to 20 DAA, stay steady from 20 to 30 DAA and then decrease up to 50 DAA, presenting an steady state from 50 to 60 DAA.

Likeness between SEPs can be measured and tested using the Pearson's correlation coefficient, r. Table 1 presents the estimated values of r, $\hat{r}$, as well as the values of the probability of null association, p-values and the 95% Confidence Intervals for the true value of r for the relevant pairs of SEPs presented in Figure 2.

TABLE 1. Correlation analyses of expression profiles for two genes in two genotypes.

							r 95% CL	
Case	Gene 1	Gene 2	Acc. 1	Acc. 2	$\hat{r}$	p-value	Lower	Upper
1	Kinesin-4	HMGB prot. 6	AS	AS	0.99778	4.6×10^{-7}	0.98433	0.99969
2	Kinesin-4	HMGB prot. 6	QU	QU	0.96014	6.0×10^{-4}	0.74767	0.99429
3	Kinesin-4	Kinesin-4	AS	QU	-0.05167	0.91239	-0.77459	0.72978
4	HMGB prot. 6	HMGB prot. 6	AS	QU	-0.15212	0.74473	-0.81214	0.67868
5	Kinesin-4	HMGB prot. 6	AS	QU	-0.10855	0.81680	-0.79650	0.70188

In Table 1 we can corroborate that the numeric values of r faithfully reflect the linear relation on time between SEPs that was observed in Figure 2. Only SEP pairs of the two different genes in the same genotype (Cases 1 and 2) have a highly positive correlation, $\hat{r} \approx 1$, with highly significant p-values. The relations of SEPs for "Kinesin-4" and "HMGB prot. 6" in genotype AS (red and orange lines in Figure 2, case 1 in Table 1) have $\hat{r} = 0.99778$ with a very small p-value, while the relation between those two genes in genotype QU (blue and green lines in Figure 2, case 2 in Table 1) have $\hat{r} = 0.96014$ also with a very small and significant p-value.

In contrast, correlations between the same gene in different genotypes, i.e., cases 3 and 4 in Table 1, have slightly negative values of $\hat{r}$, which are not significant, and the same happens for the case 5 in Table 1, which measured correlation between Kinesin-4 in AS (red line in Figure 2) with HMGB prot. 6 in QU (green line in Figure 2).

The example presented demonstrates that correlation analysis can detect significant relations between two given genes, even if those two genes have very different time expression profiles in different genotypes. We will see that the core of our method, the "*Gene2Gene*" algorithm, depends of finding high and significant correlations between pairs of genes which are repeated in various independent sources.

SI.2.2. **Robustness of a relation between genes.** Assume that we have a large number of independent genotypes, say "a", fairly representing the genetic diversity of a species of interest. Also assume that we have estimated time expression profiles for all genes in the genome, and have calculated all pairwise Pearson's correlation coefficients between all pairs of time expression profiles for different genes, $i, j;\ i \neq j$, in each one of the a genotypes: $k = 1, 2, \cdots, a$; say $\hat{r}_{ijk}$. Using the approximate distribution of $\hat{r}$ (Reverter and Chan, 2008) via the t-test, we can calculate the probability of Error Type I, p, associated with the null hypothesis "$\mathcal{H}_0 : \rho_{ijk} = 0$" versus the alternative "$\mathcal{H}_a : \rho_{ijk} \neq 0$", where ρ_{ijk} is the true value of the correlation coefficient estimated by $\hat{r}_{ijk}$.

Given that we are going to be performing many estimations of $\hat{r}_{ijk}$, we need to transform the raw p values obtained from the test into q values with the method of FDR proposed in Benjamini and Hochberg (1995). For a pair of genes $i, j;\ i \neq j$ we will reject the null hypothesis "$\mathcal{H}_0 : \rho_{ijk} = 0$" in favor of the alternative "$\mathcal{H}_a : \rho_{ijk} \neq 0$" only when $q \leq Q$, where Q is the FDR fixed by the researcher; for example fixing $Q = 0.05$ we will have a 5% expected FDR.

Given that we are looking for significant gene relations ($\rho_{ijk} \neq 0$) in "a" independent genotypes accepting a FDR equal to Q, the probability of rejecting the null hypothesis in exactly x of the a independent genotypes follows a binomial distribution, say

$$P[X = x|a, Q] = \frac{a!}{x!(a-x)!} Q^x (1-Q)^{a-x}$$

where Q represents the probability of "success", i.e., the probability of detecting a significant relation between two genes in exactly x genotypes, having tested their expression profiles in a total of "a" genotypes and applying a FDR threshold of $100 \times Q$ (FDR given here in percentage).

We are now able estimate the probability of an error in the determination of a gene relation, by adding the probabilities of positively detecting such relation in at least x independent data sources (genotypes or accessions). The error probability in the determination of a relation between any pair of different genes, say $e(x, a, Q)$, will be defined as

$$e(x, a, Q) = \sum_{i=x}^{i=a} P[X = i | a, Q] \quad (1)$$

Note that $e(x, a, Q)$ can be calculated *a priori*, i.e., before performing any experiment, and it depends only on the number of **independent** genotypes that will be explored, a, the proportion of FDR that the researcher is willing to accept, Q, and in choosing the minimum number of genotypes, $x \leq a$, where the relation will show a FDR less or equal to Q. This test is similar to the exact binomial test between two independent proportions (Laurencelle, 2021), except that we use only one binomial proportion instead of two. As numeric examples Table 2 shows the values of $e(x, a, Q)$ for some values of Q (0.05, 0.01 and 0.005) and for values of x equal to $2, 3, 4, 5$ and $x = 12$ when there are data available for $a = 12$ independent genotypes.

Table 2. Value of $e(x, a, Q)$ for different values of Q (FDR) and x (Minimum number of genotypes required) for the case where $a = 12$ independent genotypes are available.

Q	x (minimum number of genotypes)					
(FDR)	2	3	4	5	$\cdots$	12
0.100	0.34100	0.11087	0.02564	0.00433	$\cdots$	1×10^{-12}
0.050	0.11836	0.01956	0.00226	0.00018	$\cdots$	2.44×10^{-16}
0.010	0.00617	0.00021	4.64×10^{-06}	7.47×10^{-8}	$\cdots$	1.00×10^{-24}
0.005	0.00160	2.66×10^{-5}	3.00×10^{-7}	2.40×10^{-9}	$\cdots$	2.44×10^{-28}

In each row of Table 2 we see that the error, $e(x, a, Q)$, for fixed values of $a = 12$ and $Q = 0.05, 0.01$ and 0.005 (in the three rows of the table, respectively), exponentially decreases as function of x. This makes sense, because we will expect that, if a gene relation surpassed the threshold Q in a single genotype by pure chance, that event will not be repeated in other independent genotypes. Thus, the concurrence of a high and significant correlation in a large proportion of the independent genotypes will be extremely small when x approaches to a, i.e., when we repeatedly observe that the relation is found in different and independent data sources. "*Salsa*" function "`pred.error.g2g(n.acc, tot.acc, q)`" implements the calculation of $e(x, a, Q)$ (equation (1) above).

The rational core to calculate $e(x, a, Q)$, is the assumption of statistical independence between the group of genotypes employed in the study, as well as the premise that the sample of genotypes represents all relevant transcriptomic diversity in the species studied. An example where the assumption of independence of genotypes will be clearly violated, is the case where all genotypes sampled belong to full siblings of the same cross. In that case all correlations between expression profiles will be highly influenced by the alleles present at each genotype, which are a sample of the parent's alleles and not an unbiased sample of all alleles of the species of interest.

On the other hand, calculation of $e(x, a, Q)$, as exemplified in Table 2, are only valid for the transcriptomic diversity sampled by the a genotypes employed, and as said above, assuming that such set of genotypes could be considered genetically and statistically independent. For example, assume that only highly different domesticated genotypes are studied. In that case we can reasonably assume that they are "independent" because the common ancestor of them is likely to be many generations above the current domesticated genotypes. However, we could not assume that the gene relations found will also be valid for wild (non-domesticated) members of the specie. In contrast, if the collection of the a genotypes

studied includes both, domesticated as well as wild genotypes, and such collection can be considered to be an unbiased sample of all transcriptome diversity present in the specie, and the GFN is constructed by including only genes with $e(x = a, a, Q)$ (the minimum error possible), that GFN will be very likely to represent a true causal networks of genes.

Note that a natural "robustness measure" for a gene relation can be defined as the arithmetic inverse of the error probability, say $R(x, a, Q) = 1/e(x, a, Q)$. The robustness measure $R(x, a, Q)$ can take values from a minimum of 1 –when we accept that any relation appearing a single time in the a genotypes –in that case the error $e(x, a, Q) = 1$, up to a very large value when a is large and $x = a$, and thus $e(x, a, Q) \approx 0$. $R(x, a, Q)$ has a straightforward interpretation as the odds of the likelihood of obtaining relation between genes by chance, under the particular framework defined by the number of genotypes studied, "a" and the expected proportion of FDR that the researcher is willing to accept, "Q". When a and Q are already set, we can simplify the notation from $R(x, a, Q)$ to $R(x)$.

For example, assume that we have $a = 12$ genotypes and $Q = 0.05$. Then we will center our attention in $R(x) = R(x, a = 12, Q = 0.05)$. For that case, the first row in Table 2 give results of $R(x)$ for $x = 2, 3, 4, 5$ and 12. Before constructing a GFN, we need to decide how a priori how stringent we want to be to accept gene relations (edges) in our GFN. If we take relations that are repeated in only $x = 2$ genotypes, we find that $R(2) = 1/0.11836 \approx 8$, and thus the odds of a gene relation being found by chance are approximately $1 : 8$. The rounded values for $x = 3, 4$ and 5 of $R(x)$ are $R(3) = 51$, $R(4) = 447$ and $R(5) = 5436$, while for $x = 12$ we have that $R(12) \approx 4.1 \times 10^{15}$; an enormous number, equivalent to more than 4,000,000 billions. Expressing the previous numbers as odds we have $1 : 51$, $1 : 447$, $1 : 5436$, $1 : 4.1 \times 10^{15}$ for $x = 3, 4, 5$ and 12, respectively, thus the researcher can decide which value of x to select to construct a particular GFN.

In general, GFN constructed with a large value of x will have less genes than the ones constructed with a small value of x, but in counterpart we could be more confident that a GFN with a large value of x will contain genes which represent causal relationships.

SI.2.3. **The "*Gene2Gene*" algorithm.** The *Gene2Gene* algorithm can be applied when there are Standardized Expression Profiles or "SEPs" for all genes in a set of "a" independent genotypes of the species of interest. SEPs are time expression profiles with mean 0 and standard deviation 1, that summarize RNA-Seq or micro-arrays results, containing all relevant information about the significant changes of expression during the time period sampled –see details in (Escoto-Sandoval et al., 2021a; Martinez, 2022). In the cases when a gene did not change its expression significantly during the time period sampled, the SEP vector will be formed only by zeroes in all their elements, and evidently its standard deviation will be 0 and not 1 as for other SEPs. We call those SEPs "*null SEPs*". It is important to remark that any set of time expression profiles can be expressed as SEPs, thus the algorithm is applicable to any time course experiment which used a representative set of genotypes (Martinez, 2022)

Gene2Gene consists of two basic steps, say, the core estimation –implemented in the "*Salsa*" function "`core.g2g`", and the filtering step –implemented in the "*Salsa*" function "`g2g`". For the core estimation we need a set of k gene identifiers, and the set of no null SEPs for those k genes in each one of the a genotypes. For each one of the a genotypes and every one of the $n = k(k-1)/2$ different pairs of gene identifiers within genotype, the core estimation step estimates a data frame, `res.corr`, with variables: "`acc`", "`id1`", "`id2`", "`r`", "`p.r`", "`m.a`" and "`r.pos`". The next list describes each one of the variables in the data frame output by the core estimation step.

The core estimation step (function "`core.g2g`" in "*Salsa*")

- `acc` **:** The key identifier for the genotype.
- `id1` **:** The numeric identifier for the first gene in the gene pair, within the genotype `acc`.
- `id2` **:** The numeric identifier for the second gene in the gene pair, within the genotype `acc`. Note that in all cases `id1` $\neq$ `id2`.

`r` **:** Estimated value of Pearson's correlation, $\hat{r}$, between the SEPs of the genes with identifiers `id1` and `id1` in genotype `acc`.

`p.r` **:** Estimated p-value for the test of the null hypothesis $r = 0$.

`m.a` **:** Maximum of the absolute differences between the SEPs of the genes with identifiers `id1` and `id1` in genotype `acc`; the statistic $\hat{m}_a$.

`r.pos` **:** A logical variable denoting if $\hat{r} > 0$.

Note that, in the absence of null SEPs, the total number of rows in the `res.corr` will be $r = an = ak(k-1)/2$, and this number increases almost quadratically with the number of genes analyzed, k. For example, for $a = 12$, $k = 10, 100, 500$ we will have 540, 59400 and 1497000 rows in `res.corr`, respectively.

On the other hand the statistic m_a for two SEPs, say $S_1 = (s_{11}, s_{12}, \cdots, s_{1t})$ and $S_2 = (s_{21}, s_{22}, \cdots, s_{2t})$ is defined as the maximum of the absolute values of the differences between those vectors, say

$$\hat{m}_a = \hat{m}_a(S_1, S2) = \max\left(|s_{11} - s_{21}, s_{12} - s_{22}, \cdots, s_{1t} - s_{2t}|\right)$$

The objective to estimate $\hat{m}_a$ is to detect potential outliers that could be increasing the value of $\hat{r}^2$.

The second step of the *Gene2Gene* algorithm, i.e., the filtering step, consists in selecting relevant results from the output of the core data frame, `res.corr`. The input of the filtering step is presented in the next list.

`core` **:** A data frame obtained by the core step, as "`res.corr`" previously described.

`min.fdr` **:** A threshold for the False Discovery Rate (FDR) in proportion; for example if `min.fdr` = 0.05 only results having an expected FDR of 5% or less will be selected to appear in the output.

`min.r2` **:** A minimum value for the $\hat{r}^2$. For example, if `min.r2` = 0.7 only values of $\hat{r}^2 > 0.7$ will be selected. In that case, both positive values with $\hat{r} > 0.83666$ or negative values with $\hat{r} < -0.83666$ will surpass the threshold

`m.a.quan` **:** A quantile threshold for the distribution of the statistic $\hat{m}_a$. For example, if `m.a.quan` = 0.05, the extreme values in the 5% left hand side and right hand side of the $\hat{m}_a$ distribution will be filtered from the output to discard potential outliers.

`n.min.acc` **:** Number of genotypes where the results must be repeated to be present in the output.

The next list enumerates the filtering step of the *Gene2Gene* algorithm.

The filtering step (function "`g2g`" in "*Salsa*")

(1) Calculate the q-values as `q.r`, from the p-values (`p.r`) in the `core` data frame using the algorithm in (Benjamini and Hochberg, 1995).

(2) Eliminates from `core` the rows which have `q.r` $\geq$ `min.fdr`. This let's in `core` only cases where the FDR threshold is fulfilled.

(3) Segregates the current data frame `core` into two data frames, say `x.rp` and `x.rn` which contain values of $\hat{r} > 0$ and $\hat{r} < 0$, respectively.

(4) A value `quan.r.pos` is calculated by the R function "`quantile`" with arguments `x.rp$m.a` (the values of the $\hat{m}_a$ statistic in data frame `x.rp`), and `probs = 1 - m.a.quan`.

(5) Values of `x.rp` are filtered to fulfill the condition `x.rp$m.a` $\leq$ `quan.r.pos`. This eliminates the `m.a.quan` proportion of the rows of `x.rp` having the largest values of $\hat{m}_a$.

(6) A value `quan.r.neg` is calculated by the R function "`quantile`" with arguments `x.rn$m.a` (the values of the $\hat{m}_a$ statistic in data frame `x.rn`), and `probs = m.a.quan`.

(7) Values of `x.rn` are filtered to fulfill the condition `x.rn$m.a` $\geq$ `quan.r.neg`. This eliminates the `m.a.quan` proportion of the rows of `x.rp` having the smallest values of $\hat{m}_a$.

(8) A variable, `x.rp$edge.tag`, is created in the `x.rp` data frame by pasting `x.rp$id1` with `x.rp$id2`, separated by "`-`". This creates a unique tag for each one of the gene pairs related by $\hat{r} > 0$.

(9) Variable `x.rp$edge.tag` is tabulated, and only cases of gene pairs that surpass the parameter `n.min.acc` are let into data frame `x.rp`.

(10) A variable, `x.rn$edge.tag`, is created in the `x.rn` data frame by pasting `x.rn$id1` with `x.rn$id2`, separated by "`-`". This creates a unique tag for each one of the gene pairs related by $\hat{r} < 0$.

(11) Variable `x.rn$edge.tag` is tabulated, and only cases of gene pairs that surpass the parameter `n.min.acc` are let into data frame `x.rn`.

(12) A data frame, `win.cand`, is created by binding by rows the `x.rp` and `x.rn` data frame.

(13) A character vector, `tag`, with all unique values of the variable `x.rn$edge.tag` is created.

(14) A new data frame, `winners`, is created to contain relevant statistics for each one of the relevant gene pairs that passed all filters, and which are identified by the values of `tag`. The number of winners, i.e., the length of `tag`, is denoted here as w.

(15) For each group of rows of `win.cand` defined by the values of variable "`tag`", the algorithm calculates the variables of each one of the gene pair "*winners*" in a data frame called "`winners`", which is described in the next list:

`w.id` : Numerical identifier for the "winner" (values $1, 2, \cdots, w$).

`tag` : Tag that identifies the winner.

`r.pos` : Logical. Is $\hat{r} > 0$?

`id1` : Numerical identifier of the first member of the gene pair.

`id2` : Numerical identifier of the second member of the gene pair.

`n.acc` : Number of genotypes in the winner pair (note that this number will be always $\geq$ `n.min.acc`).

`avg.r` : Average value of $\hat{r}$ in the winner.

`avg.p.r` : Average value of p-values in the winner.

`avg.q.r` : Average value of q-values in the winner.

`avg.m.a` : Average value of $\hat{m}_a$ statistic in the winner.

`l.acc` : Character variable containing the genotype keys included in the winner.

(16) Finally the algorithm output the "winners" data.frame (`winners`) as well as intermediate results, and could also include descriptors of the genes included in the winners set, even when this is not strictly necessary.

Note that the core estimation step (implemented in function "`core.g2g`" in "*Salsa*") needs as variable parameter only a set of gene identifiers, which will usually be formed by genes that share annotations about a particular biological process (BP), or some other aspect that is of interest and that could result in what we call a Gene Functional Network (GFN) –but could also be, for example, "A mitochondrion related network", if genes related with that organelle are selected, etc.

In contrast, the filtering step (implemented in function "`g2g`" in "*Salsa*") includes in the input the product of the first step (core estimation) but also a set of thresholds which will limit the number of gene pair "winners", i.e., the number of relations that will be considered biologically relevant.

While the core estimation needs to be run only once for a given set of genes identifier, the filtering step can be run more than once if the user wants to make a sensitivity analysis with different sets of thresholds to obtain an optimum result for the process of interest.

The main parameter for the filtering step is the number of genotypes that is needed to ratify a relation –the parameter `n.min.acc` in function `g2g`, which can vary in the natural numbers $1, 2, \cdots, a$ –"a" being the number of genotypes available for scrutiny. As can be seen in Table 2, there is little point in running the algorithm with values of `n.min.acc` ≤ 3, because in those cases many of the relations found will be too weak to be taken into account. As it could be expected, in general the number of "winners" –relations ratified, increases rapidly as the parameter `n.min.acc` decreases from its maximum value of a. This is due to the fact that some of the relations found with, say `n.min.acc`$= a - 1$ will be particular to one of the genotypes not included. In contrast, when `n.min.acc`$= a$ all the relations found are ratified by all available genotypes, making the GFN the most robust that can be estimated for the set of genes of interest.

On the other hand, the remaining parameters in the filtering step, say, `min.fdr`, `min.r2`, `m.a.quan` and `n.min.acc`, govern the stringency of the correlations that will be included into the winner's set. In the case of our chili pepper fruit experiment (see Results), we empirically found that when asking for the maximum number of genotypes (`n.min.acc`$= 12$), reasonable and biologically relevant results where obtained by setting the filtering parameters to `min.fdr` $= 0.1$, `min.r2` $= 0.7$ and `m.a.quan` $= 0.05$ Thus, we are not too stringent with the quality of the correlations, but ask that such correlations will be found in all available genotypes.

The same procedure described here for the *Gene2Gene* algorithm can be employed to look for Transcription Factors (TF) of a gene of interest. We call this approach the "*Gene2TF*" algorithm, and it is implemented in the "*Salsa*" function "`g2g.TFcandidates`". Briefly, the "*Gene2TF*" needs only the identifier of a single "target gene", say "`tg.id`" and the same parameters than the filtering step in the *Gene2Gene* algorithm. It analyzes the gene-wise correlations of the SEP of the target gene with each one of the SEPs of genes annotated as TF in *Capsicum*, doing this for all genotypes available. The output consists on a set of TF that resemble the time expression profiles of the target gene in as many genotypes as considered relevant and with stringency dictated by the user.

SI.2.4. **A general constructive approach.** The *Gene2Gene* algorithm recovers gene relations that are repeated in a set of independent genotypes, allowing the recovery of robust GFN. In principle, one could attempt to construct a network with all the genes in the genome and all genotypes available. Additionally to the high computational cost of implied in the estimation of such whole network, it will almost surely present the "hairy ball" effect, making its direct interpretation very difficult. Also, it will include not identified and not annotated genes which will complicate interpretation even further. Moreover, if we ask such general network to contain only relations repeated in many or all genotypes, we will be unable to detect the degree of conservation of the relations in different genotypes.

Instead of attempting to estimate a general network, we propose to orderly estimate GFN modules which will contain only genes annotated into a particular biological process (BP). The selection of the particular BP will respond to the particular interest of the research group. This allows the researcher to evaluate the robustness of each GFN module, to elucidate if groups of genotypes present interesting functional differences, or all gene relations are shared among the whole genotype set. On a second step, two or more GFN modules could be linked to obtain a more general panorama of the biological processes studied and how they relate to each other.

SI.2.5. **Linking GFN modules.** After having estimated GFN for various biological processes (BP) of interest, we can see if such GFN are independent, i.e., if they do not have common genes, or if there are genes shared between pairs of them. In that late case, we can construct a "Meta Network" (MN) by linking al pairs of GFN by the genes that are shared between pairs. It is important to realize that MN are automatically annotated by the fact that genes between pairs of GFN are shared, i.e., if GFN

"A", "B" and "C" form a MN by having common genes between "A" and "B", "B" and "C" and "A" and "C", then we can consider the MN as the union of the three original GFN. Also, the strength of the linking between pairs of GFN can be judged by the number of genes shared between each pair.

Other possibility is that all genes and relations in the GFN "B" are also included into the GFN "A". In that case we will consider "B" just a subset of "A". Also more complex set relations can appear in a MN, and a relevant fact is that set theory can be applied to study such relations, which will reflect in part the complexity existent in the original ontology used to annotate the genes, but has also the potential to discover new and unexpected relationships.

SI.3. Data analyses

To demonstrate our approach we use data from a large experiment comprising gene expression during fruit development in chili pepper (*Capsicum annuum* L.) in 12 different genotypes, 6 domesticated, 4 wild and 2 crosses (Escoto-Sandoval et al., 2021a). Gene expression was estimated every 10 days, from mature flower until fruit maturity at 60 days after anthesis (DAA). For each gene within each genotype, time expression profiles are summarized as Standardized Expression Profiles (SEPs), which take into account significant expression changes in neighboring time intervals. SEPs allow the study different angles of gene expression, by comparing groups of genes with different annotations or from different origin (Martínez et al., 2021; Escoto-Sandoval et al., 2021b; Martinez, 2022). All data and functions for analysis are publicly available for the R environment (R Core Team, 2013) in the package "*Salsa*" (Martínez and Escoto-Sandoval, 2021), which current version (1.0) includes all functions related to the *Gene2Gene* and *Gene2TF* algorithms used here.

We present the estimation of three GFN, using genes annotated in the Biological Processes (BP), "*cell cycle*" (GO:0007049), "*reproduction*" (GO:0000003) and "*response to virus*" (GO:0009615).

Because we want to present highly robust GFN, in all three cases we selected gene relations that are corroborated in all the 12 genotypes, and thus have a very small probability ($\approx 1 \times 10^{-12}$; see Methods) of the associations happening by pure chance. Given that we are highly stringent by asking the relations to be repeated in the 12 genotypes, we used relatively less stringent parameters for the individual pairwise relations, fixing a minimum of FDR of 10% (`min.fdr=0.1`), a minimum of $\hat{r}^2$ (`min.r2=0.7`) –thus relations must explain at least 70% of the variance of gene expression patterns and the absolute value of $\hat{r}$ must be at least 0.83666 and eliminating the 5% (`m.a.quan=0.05`) of the most extreme values of the maximum absolute statistic, m_a, and thus eliminating potential regression outliers.

Individual GFN for cell cycle, reproduction and response to virus, are denoted here as "`celcy`", "`rep`" and "`vir`", respectively. The R calculations to obtain those GFN are shown in Appendix SI.5.1. To ease presentation, we begin by showing and summarizing the plots of each one of the GFN without discussing details. All figures of networks were obtained using the R package "*igraph*" (Csardi and Nepusz, 2006), however, for brevity we do not present here the R code to obtain such figures. In all the network plots, structural genes are represented by circles while Transcription Factors (TF) are represented by squares, and genes are labeled with a unique number, presented in Table 6 in the Appendix. Different colors are used to plot each GFN.

SI.3.1. GFN for three biological processes (BPs).

SI.3.1.1. *GFN for "*cell cycle*" (`celcy`) genes.* See Appendix SI.5.1 `*celcy` for R code to obtain this GFN.

To estimate the `celcy` GFN, the *Gene2Gene* algorithm began with a set of 352 genes consistently expressed in the 12 genotypes and annotated in the BP "cell cycle". 81 gene pairs surpassed the parameters thresholds, forming a network that includes 29 different genes. Figure 6 panel (A) presents the GFN for the "cell cycle" (`celcy`) BP.

In Figure 6 (A) we can see that the cell cycle (`celcy`) GFN is constituted by 4 independent components (disconnected graphs). This means that there are 4 highly different expression time time profiles for the genes annotated in this BP. Beginning from the upper left hand side corner of the panel, and advancing clock-wise, we find that the first component includes 4 genes –one of those is a TF and 4 connections. The second one (top right hand side corner) includes only two genes, and one of these is a TF. The third one (bottom left hand side corner) is the most complex of the four, including 13 genes, 5 of which are TF. The fourth (bottom right hand side corner) includes 10 genes –none of which is a TF.

SI.3.1.2. *GFN for "reproduction" (`rep`).* See Appendix SI.5.1 `*rep` for R code to obtain this GFN.

To estimate the `rep` GFN, the *Gene2Gene* algorithm began with a set of 228 genes consistently expressed in the 12 genotypes and annotated in the BP "reproduction". 29 gene pairs surpassed the parameters thresholds, forming a network that includes 10 different genes. Panel (B) in Figure 6 presents the GFN for the "reproduction" (`rep`) BP.

In Figure 6 (B) we can see that the GFN `rep` consists of two independent components. The first component (upper left hand side corner in the figure) includes only two genes, one of which is a TF. The second (bottom left hand side corner) includes 8 genes, two of which are TF, and the whole network has 28 connections (edges).

SI.3.1.3. *A GFN for "response to virus" (`vir`).* See Appendix SI.5.1 `*vir` for R code to obtain this GFN.

To estimate the `vir` GFN, the *Gene2Gene* algorithm began with a set of 33 genes consistently expressed in the 12 genotypes and annotated in the BP "response to virus". 6 gene pairs surpassed the parameters thresholds, forming a fully connected network that includes 4 different genes. Figure 6 (C) presents the GFN for the "response to virus" (`vir`) BP.

In Figure 6 (C) we can see that the graph for the GFN `vir` is fully connected, i.e., each one of the four genes are connected with the other tree, and one of them (the one with the identifier 34) is a TF.

SI.3.1.4. *Strength of the GFN estimated (`celcy`, `rep` and `vir`). A priori*, we know that all 3 estimated GFN are highly robust, because the relations found are repeated in all 12 genotypes, having a very small probability of happening by chance, $\approx 1\times10^{-12}$. However, we employed relatively low stringency in the filtering step of the algorithm; in all 3 cases those thresholds were a FDR of 10% (`min.fdr=0.1`), a minimum of $\hat{r}^2 = 0.7$ (`min.r2=0.7`) and the extreme 5% of the $\hat{m}_a$ values were excluded (`m.a.quan=0.05`).

It is important to remember that each one of the relations found is estimated in a total of 12 independent genotypes, thus for each one of the relations we have 12 values of $\hat{r}$, $\hat{m}_a$, as well as the corresponding 12 values of the probability, p, from the correlation tests, which are converted into q-values by the (Benjamini and Hochberg, 1995) algorithm to evaluate FDR.

Table 3 presents some statistics to judge the general strength of the correlations estimated in the three GFN, while Figure 3 presents the distributions of the average values of $\hat{r}$ in each one of these GFN.

Table 3. Statistics for gene relations within each GFN.

		Worst case:			Medians:		
GFN	n relations	min ($\hat{r}$)	max ($\hat{m}_a$)	max ($\hat{q}$)	med($\hat{r}$)	med($\hat{m}_a$)	med($\hat{q}$)
`celcy`	81	0.95523	0.46155	0.03450	0.98536	0.25104	0.00674
`rep`	29	0.98143	0.32394	0.01252	0.99481	0.13979	0.00121
`vir`	6	0.98686	0.29160	0.00283	0.99441	0.15444	0.00095
All	116	0.95523	0.46155	0.03450	0.99441	0.15444	0.00121

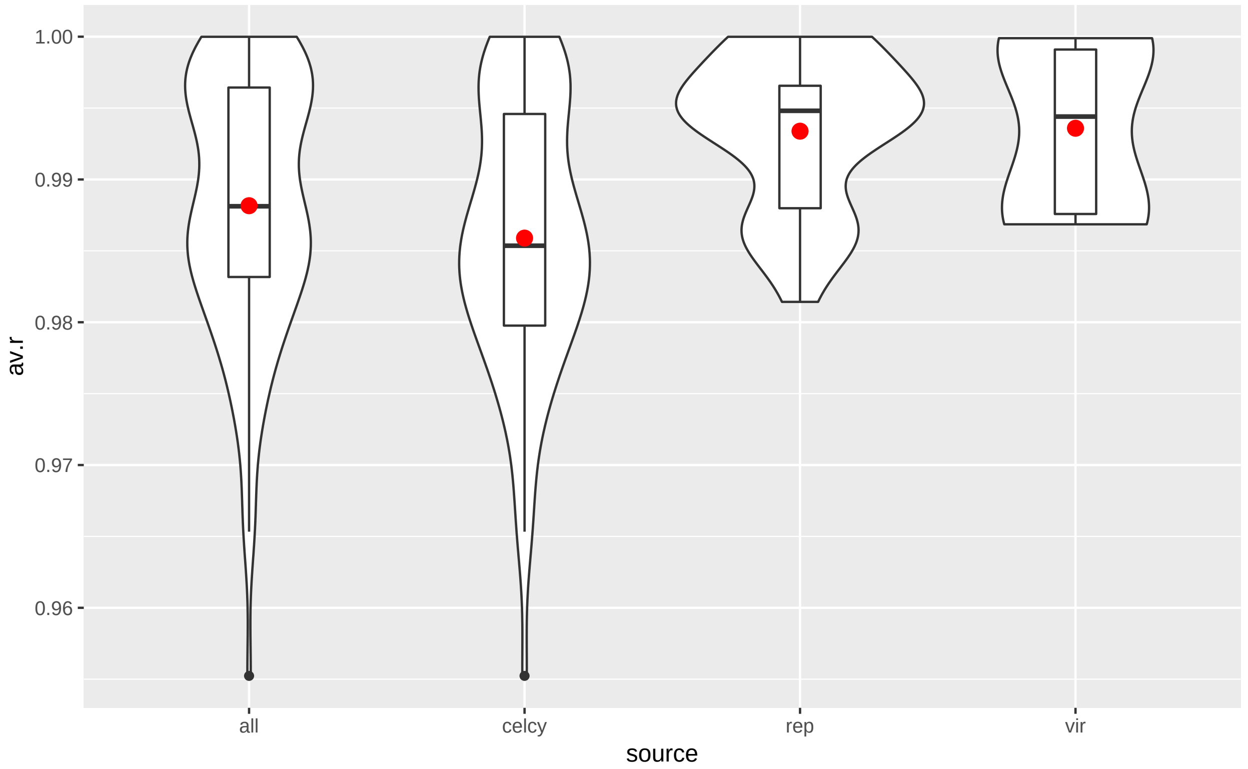

FIGURE 3. Distributions, as violin plots including box plots and means as red circles, for the estimated values of the averages Pearson's correlation coefficients, $\hat{r}$. Y-axis: average values of $\hat{r}$ (avg.r); X-axis: source. Distributions include 116, 81, 29 and 6 average $\hat{r}$ estimated for "all", "celcy", "rep" and "vir", respectively.

In Table 3, columns grouped in the "*Worst case*" section, present the values that are closer to the thresholds set by the run of the algorithm in each one of the GFN. From these values we can see that, even when the threshold for $\hat{r}^2$ was `min.r2=0.7`, corresponding to positive values of $\hat{r} = \sqrt{.7} \approx 0.84$, the *minimum* average value of $\hat{r}$, 0.95523, is far away to the limit imposed by the threshold, 0.84 This implies that the method is obtaining values that are very close to the maximum of $r = 1$, in almost all the cases.

On the other hand, the distributions of the average values of $\hat{r}$ presented as violin plots in Figure 3 show that in very few cases the average of $\hat{r}$ was below of 0.97, and that the relations in the GFN "`rep`" and "`vir`" have a distribution very close to the maximum value of $r = 1$, a fact that can also be collaborated by the values in Table 3. Also, the distribution of the average $\hat{r}$ in the whole set of the 116 estimated relations (first violin plot at the left hand side in Figure 3), shows an almost symmetric distribution (mean and median coincide), even when a few values are relatively smaller, causing a long but slim lower tail.

By examining the columns grouped in the "*Medians*" section in Table 3, which presents the medians for $\hat{r}$ and $\hat{q}$-values, we see that such values are far away from the corresponding thresholds of 0.84 and 0.1, being equal to ≈ 0.99 and ≈ 0.001, respectively. With reference to the m_a statistic, its maximum estimated value (worst case) was $\hat{m}_a \approx 0.46$ –less than half the standard deviations of SEPs, while its median is ≈ 0.15, implying that the relations that passed the filters are not highly influenced by regression outliers.

Furthermore, in all cases that we examined, the realized values of FDR for the relations found, say $\hat{q}$, are much more smaller than the original *a priory* threshold set, say Q –which we set to $Q = 0.1$ or 10%. We need to remember that q-values refer to the transformation of a set of p-values performed by the

algorithm presented in (Benjamini and Hochberg, 1995) with the objective to correct by multi-testing in genomic studies, and obtain a reasonable rate of "False Discoveries"; i.e., false positives. We began by setting a FDR of $Q = 0.1$, thus, all test with a value of $\hat{q} \geq 0.1$ will be discarded, granting a *maximum* or 10% of false discoveries. However, the *estimated* values of q which where considered to form part of the GFN were, in all cases, much smaller than the fixed threshold, i.e., in all cases $\hat{q} \lll Q$. This can be corroborated in Figure 4.

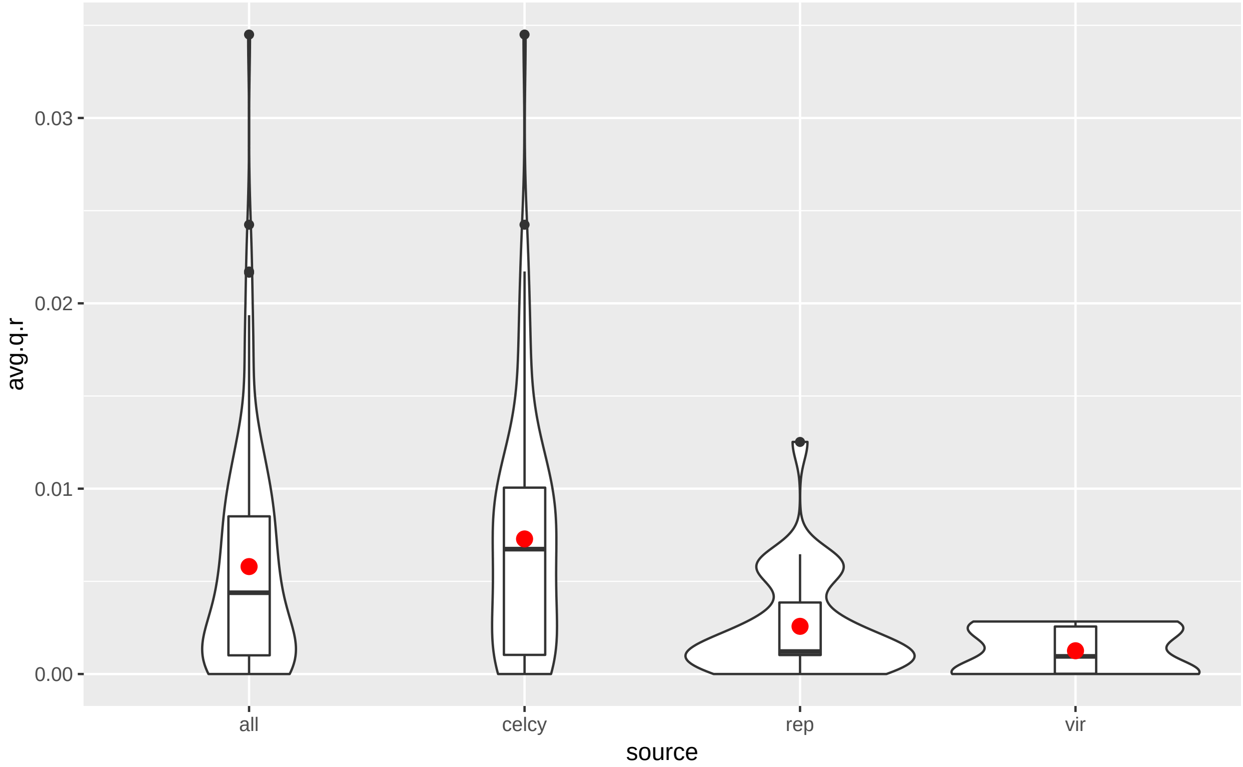

FIGURE 4. Distributions, as violin plots including box plots and means as red circles, for the estimated values of the averages of the $\hat{q}$-values when testing the Pearson's correlation coefficients, $\hat{r}$. Y-axis: average values of $\hat{q}$ (avg.q.r); X-axis: source. Distributions include 116, 81, 29 and 6 average $\hat{q}$ from the sources "all", "celcy", "rep" and "vir", respectively.

While the threshold set was `min.r2=0.1`, implying a FDR of 10%, the worst (larger) value of q was ≈ 0.03, more than three times smaller than the threshold set (the point shown as outliers, above 0.03, in the `all` and `celcy` sources in Figure 4). On the other hand, the medians of $\hat{q}$ (horizontal lines within the box plots), as well as the averages of $\hat{q}$ (shown as red circles in the distributions) are in all cases, well below 0.01 From this fact we can conclude that, with only three exceptions (outliers, shown as black points in the distributions of `celcy` and `rep`), the realized FDR was in fact smaller than 1% ($\hat{q} < 0.01$), and thus the potential error in declaring the gene relations is negligible, having a value of 1×10^{-24} (see Table 2).

In summary, we can be highly confident in that the three GFN estimated, `celcy`, `rep` and `vir`, are very likely to include mostly causal relationships between the genes involved.

SI.3.2. **Sensitivity of the *Gene2Gene* algorithm to input parameters.** Additionally to the minimum number of genotypes where the relation must be found, `n.min.acc`, that governs the robustness of the relations found, the *Gene2Gene* algorithm includes other three parameters, `min.fdr`, `min.r2` and `m.a.quan` which allow to adjust the stringency degree for the relations that will be reported. `min.fdr`

controls the maximum of the FDR by setting the minimum $\hat{q}$-value of the tests on the correlation coefficient, `min.r2` is a threshold for the minimum $\hat{r}^2$ that relations must have to be reported, and, finally, `m.a.quan` discards the upper and lower quantiles of the distribution of the $\hat{m}_a$ statistic to filter out potential regression outliers.

In all runs of the algorithm presented here, we set the values of the parameters to `n.min.acc=12` –thus asking for relations that are repeated in all genotypes available, while the remaining parameters were set to values that *a priory* appeared reasonable, say `min.fdr=0.1`, `min.r2=0.7` and `m.a.quan=0.05`.

To evaluate the sensitivity of the *Gene2Gene* algorithm to these three parameters, we defined a grid of four values for each parameter. The values in that grid are presented in Table 4, while general results are plot in Figure 5.

TABLE 4. Grid of parameter values used in the sensitivity analyses.

Parameter	Values				Direction:
`min.fdr`	0.010	0.050	0.100	0.150	More to less stringent.
`min.r2`	0.600	0.700	0.800	0.900	Less to more stringent.
`m.a.quan`	0.010	0.025	0.050	0.100	Less to more stringent.
In red: values previously employed.					

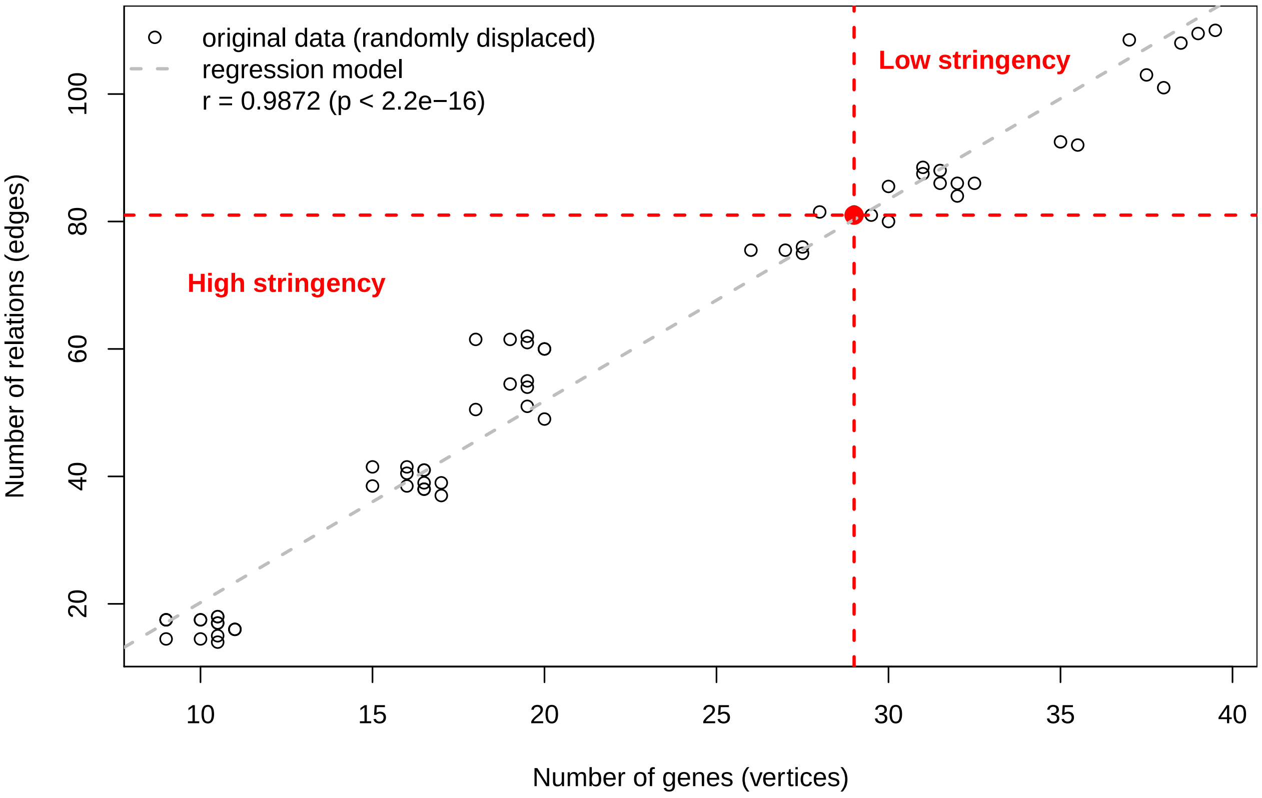

FIGURE 5. Number of genes (X) and number of relations (Y) obtained by running the *Gene2Gene* algorithm with a grid of parameters. Original data are randomly displaced in both dimensions to avoid overlapping. Red point and coordinates at values obtained with original `celcy` parameters.

The grid presented in Table 4 defines $4 \times 4 \times 4 = 64$ different parameters combinations. Those 64 parameters combinations were tried for the "`celcy`" case, measuring two values of the output, say, the number of genes, n_G (nodes), and the number of relations, n_R (edges), that resulted.

In Figure 5 we can see the general tendency of the output variables, n_G in the X-axis and n_R in the Y-axis. In that figure original output values were slightly displaced in both dimensions to avoid overlapping of the points. First, there is a strong linear relation between n_G and n_R, with a highly significant value of $\hat{r} = 0.9872$. The grey dashed line shows the regression model fitted to the output, while the red point shows the points obtained with the parameter values `min.fdr=0.1`, `min.r2=0.7` and `m.a.quan=0.05` employed in the `celcy` GFN estimation. Also, this point define quadrants in the two dimensional space $n_G \times n_R$, delimited by red dashed lines. The lower left hand side quadrant includes cases where more stringent parameter values were employed, while the upper right hand side quadrant includes cases where less stringent parameter values were set, and in general we can see that more stringent parameter values imply smaller values of n_G and n_R, while the opposite happens when less stringent parameters are employed.

For all 64 combinations of parameters, the number of genes included, n_G, varied between a minimum of 10 and a maximum of 39, with a median of 19, while the number of relations, n_R, ranged between 14 and 109, with a median of 54.

For example, the minimum value of n_R, $n_R = 14$, was found at parameters `min.fdr=0.01`, and `m.a.quan=0.1`, independently of the values of `min.r2=0.6, 0.7, 0.8, 0.9` and those points also correspond with the minimum value of n_G, $n_g = 10$. On the other side, the maximum value of n_R, $n_R = 109$, was found at parameters `min.fdr=0.15`, `m.a.quan=0.01` and `min.r2=0.6, 0.7` and those points also include the maximum value of genes, $n_G = 39$.

In general, by measuring the linear dependence of the output variables, n_R and n_G to the values of the parameters `min.fdr`, `min.r2` and `m.a.quan` explored in the grid (results not shown), we concluded that the most important parameter was `min.fdr`, followed by `min.r2`, and when these two were fixed, the values of `m.a.quan` did not alter the output very much. However, non-linear interactions between the three parameter values are important.

In summary, the *Gene2Gene* algorithm allows the estimation of robust interactions between genes, and by tuning the parameters in agreement with the researcher interest, GFN with strong correlations can be recovered.

SI.3.3. **A "*Meta Network*" (MN) linking the GFN `rep`, `celcy` and `vir`.** We have seen how to estimate very robust GFN which are backed up by results that are replicated in all 12 genotypes and thus have a very small likelihood of including artifactual relations between genes. The three GFN previously estimated are: "`rep`" for the BP "Reproduction" (GO:0000003), which includes 10 genes linked by 29 relations; "`celcy`" for the BP "Cell Cycle" (GO:0007049), which includes 29 genes and 81 relations and "`vir`" for the BP "Response to Virus" (GO:0009615) which includes 4 genes fully linked by 6 relations.

In general, distinct GFN can be linked into a Meta Network (MN) if they share one or more genes in common. This is possible precisely because gene annotation to particular BP in GO is an *ontology* (Ashburner et al., 2000; Rhee et al., 2008), i.e., an structure that is not fully hierarchical. Thus, the same gene can be annotated in various BP and this brings the opportunity to link various GFN.

The GFN previously estimated for the BPs cell cycle (`celcy`), reproduction (`rep`) and response to virus (`vir`) present some genes that are common by pairs. `celcy` and `rep` have 6 genes in common, the ones with identifiers 11478, 24186, 23951, 24093 and 6576, which correspond to vertices 9, 10, 14, 15 and 23 in all figures. `celcy` and `vir` have 2 genes in common, with identifiers 4788 and 8415, which correspond to vertices 22 and 25 while `rep` and `vir` have only the gene with identifier 8415 (vertex 25), which in fact link the three GFN together, i.e., the gene with identifier 8415 (vertex 25) belongs to the three GFN, `celcy`, `rep` and `vir`. That gene is identified as a BTB/POZ domain-containing protein DOT3 isoform X2 (see Table 6 for the full list of genes and their identifications).

Figure 7 presents different aspects of the MN formed by linking the GFN `celcy`, `rep` and `vir`.

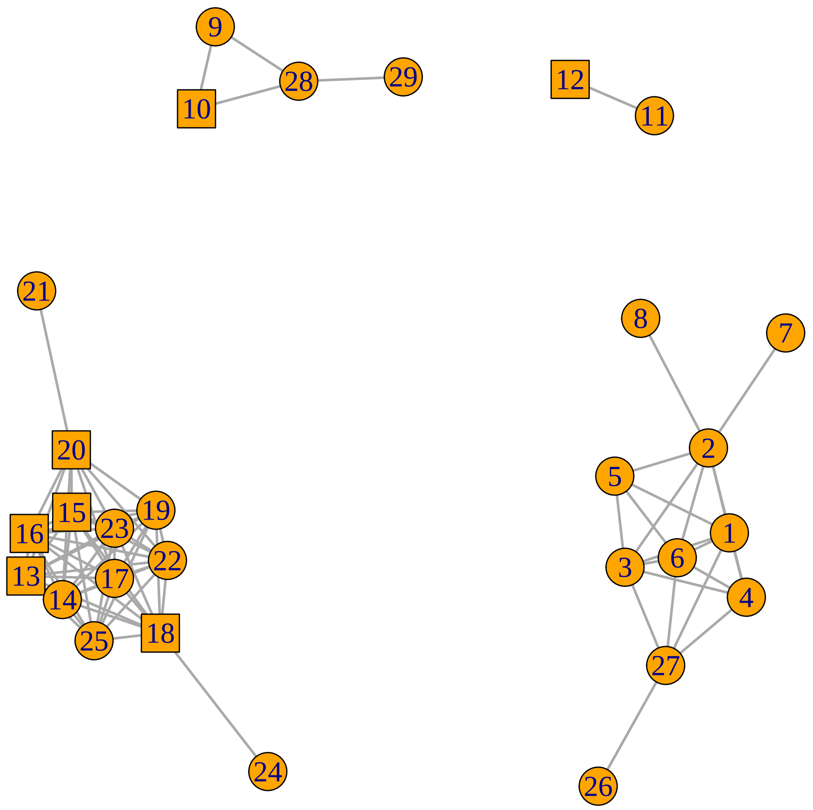

(A) GFN for "cell cycle" (`celcy`; Fig. 2 in main text).

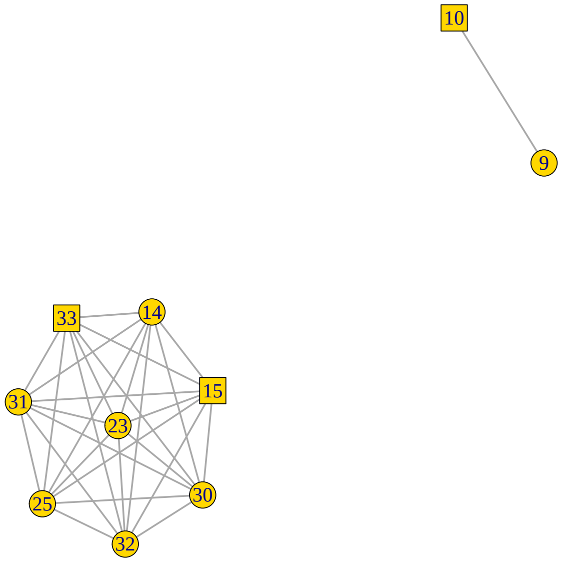

(B) GFN for "reproduction" (`rep`; Fig. 3 in main text).

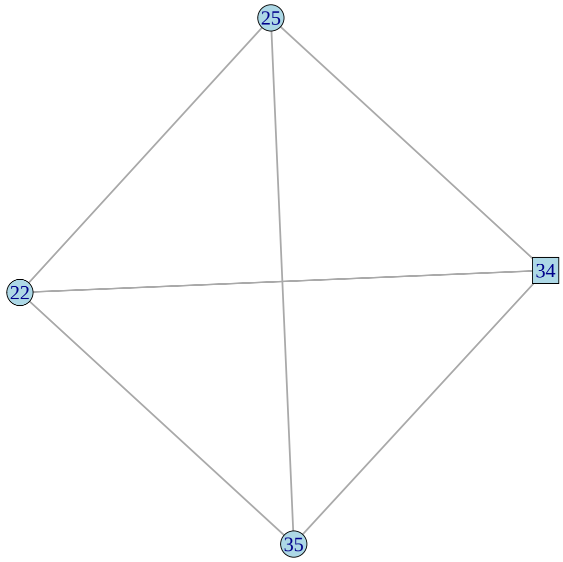

(C) GFN for "response to virus" (`vir`; Fig. 4 in main text).

FIGURE 6. Plots of GFN for "cell cycle" [`celcy`] in panel (A), "reproduction" [`rep`] in panel (B), and "response to virus" [`vir`] in panel (C). See Table 6 for gene identifiers and Appendix SI.5.1 for the estimation process in R.

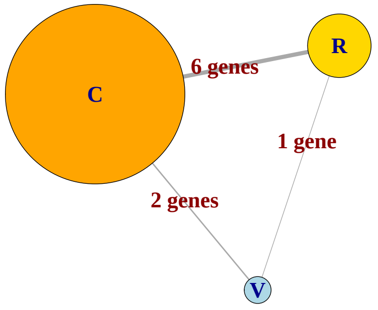

(A) MN Summary (Fig. 5 in main text).

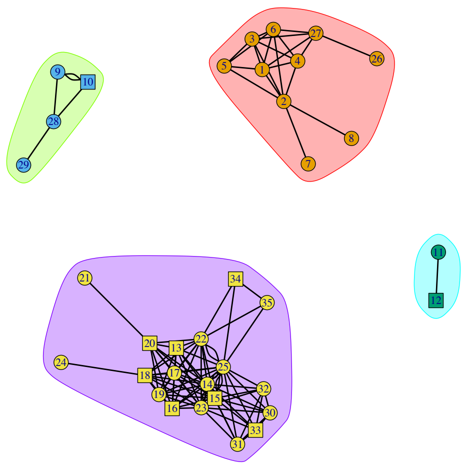

(B) MN Components.

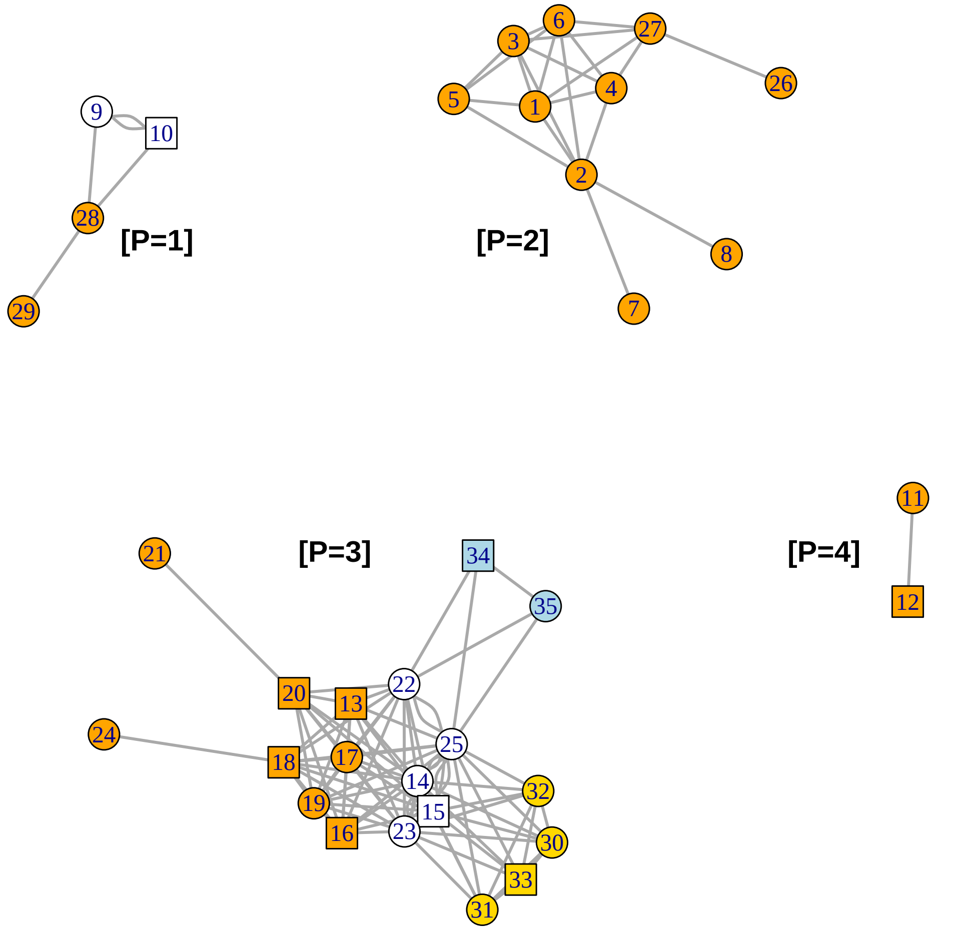

(C) Meta Network (MN; Fig. 6 in main text)

FIGURE 7. **Meta Network (MN) formed by 35 genes with 116 links.**
(A) MN Summary. In (B) and (C) structural genes shown as circles and Transcription Factors (TF) as squares. (B) shows the 4 independent components. In (C) colors of nodes denote the GFN to which the genes belong; in white genes in more than one GFN, in orange genes that belong only to cell cycle (`celcy` set C), in blue genes exclusively in response to virus (`vir` set V) and in gold yellow genes exclusively in reproduction (`rep` set R). In (C) the four different gene expression patterns are denoted by **P**. See Table 6 for gene identification.

In Figure 7 (A) we can see a summary of the MN formed by the three GFN. In that panel the nodes represent the GFN `celcy` as **C** in orange, `rep` as **R** in gold yellow and `vir` as **V** in blue. The sizes of those vertices are proportional to the number of genes, which are 29 for `celcy` (**C**), 10 for `rep` (**R**) and 4 for `vir` (**V**). On the other hand, as annotated in the panel, the width of the edges are proportional to the number of genes shared between the pairs of GFN; 6 between `celcy` (**C**) and `rep` (**C**), 2 between `celcy` and `vir` (**V**) and 1 between `rep` and `vir`. Thus, in summary panel (A) in Figure 7 shows the interconnections between the three GFN.

Figure 7 (B) shows how the MN presents 4 independent (non-connected) subgraphs. Beginning from the up left hand side corner and going clockwise, we have subgraphs formed by 4, 10, 2 and 19 genes, respectively. Those 4 subgraphs are formed because the genes in them have characteristic expression patterns that greatly differ between them.

In Figure 7 (C) we can see more clearly the 4 subgraphs. Structural genes are represented by circles, while transcription factors are represented by squares. Also, genes are colored by the GFN to which they belong. White denote genes that are shared in more than one GFN, orange genes that appear only in cell cycle (`celcy`), gold yellow genes that appear only in reproduction (`rep`) and blue only in response to virus (`vir`); the identifiers of all genes are in Table 6.

Importantly, the disconnected plots within the MN in panel (C) in Figure 7 are marked by the labels **[P=1]** to **[P=4]** because they include genes which present a highly alike expression pattern, but which expression patterns are unlike between them. Next we will examine each one of the four distinctive time expression profiles.

SI.3.4. **Distinctive time expression profiles within the MN.** We have a MN that includes 35 genes and 116 connections (panel (C) in Figure 7). However, those 35 genes form 4 disconnected graphs, which include 4, 10, 19 and 2 genes in the subgraphs labeled with **[P=1]**, **[P=2]**, **[P=3]** and **[P=4]** in that panel. Figure 8 presents the plots of the time expression profiles (SEPs) for those 4 groups of genes, while Table 5 presents the most relevant characteristics of the disconnected graphs in the MN.

Figure 8 clearly shows that the average SEPs for the genes included in the four subgraphs of the MN (**[P=1]** to **[P=4]** in panel (C) of Figure 7) present highly different patterns, and this in turn explain why the four subgraphs are completely disconnected. In panels (A) to (C) in Figure 8, corresponding to **[P=1]** to **[P=4]**, respectively, we plot not only the average SEP for the whole set of the 12 genotypes (grey line), but also the average SEPs for the 6 domesticated (D) genotypes in red and for the 4 wild genotypes (W) in blue. We did that segregation of SEPs by groups because we know that D and W genotypes present expression differences in many genes during fruit development (Martínez et al., 2021). For each one of the four cases, we tested if the differences observed between the D and W genotypes were significant via the "*Salsa*" function "`analyze.2.SEPs`", and the legend within each panel presents the corresponding p-value. Also, the plots in Figure 8 shows the 95% CI for the mean expression at each one of the times sampled.

Table 5. Characteristics of the disconnected graphs.

					Averages:		
**P**	n relations	n genes	n TF	$\hat{r}$	p-values	q-values	$\hat{m_a}$
**1**	5	4	1	0.97612	0.00060	0.01540	0.32299
**2**	21	10	0	0.98288	0.00031	0.00918	0.27402
**3**	89	19	7	0.99023	0.00012	0.00435	0.18464
**4**	1	2	1	0.97530	0.00069	0.01594	0.34914
$\Sigma =$	116	35	9	$\bar{\mathbf{r}} = 0.98$	$\bar{\mathbf{p}} = 0.0004$	$\bar{\mathbf{q}} = 0.01$	$\bar{\mathbf{m}}_\mathbf{a} = 0.3$

Note: See column "**P**" in Table 6 for the identifiers and description of genes included in each expression pattern, **P**; plots of **[P=1]** to **[P=4]** are in panel (C) of Figure 7.

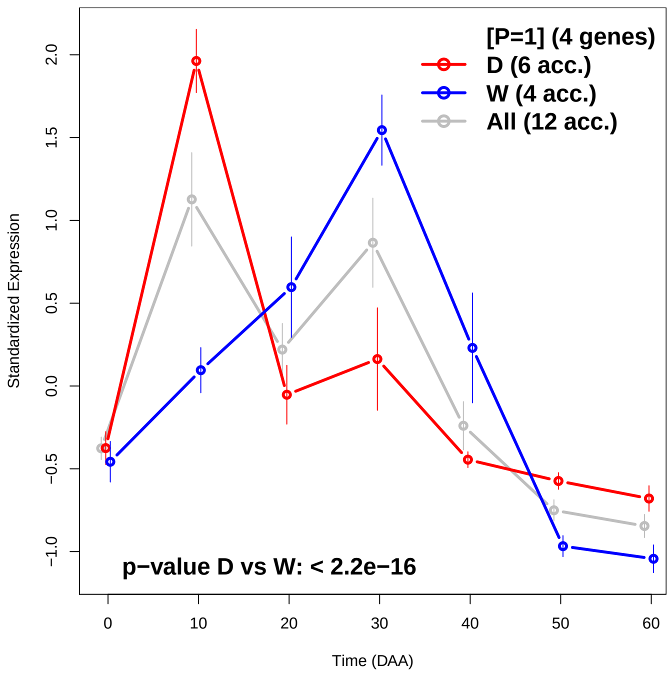

(A) Panel **(a)** in Figure 7 in main text.

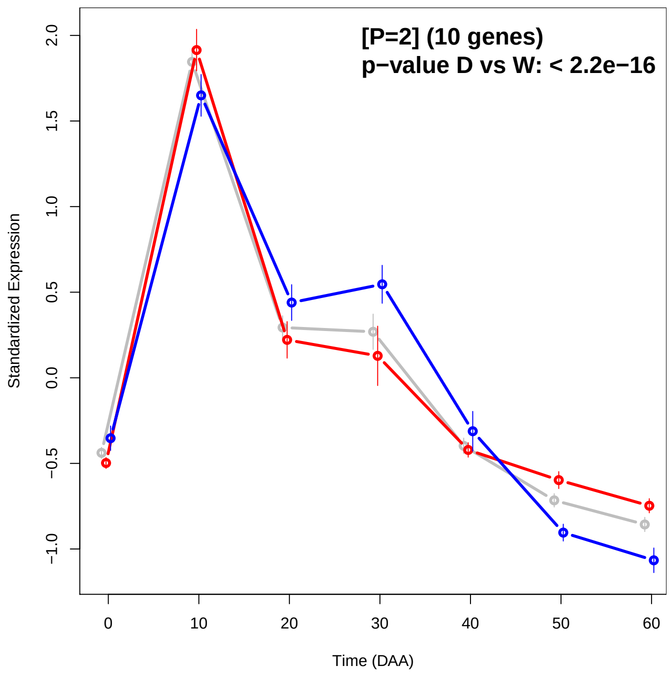

(B) Panel **(b)** in Figure 7 in main text.

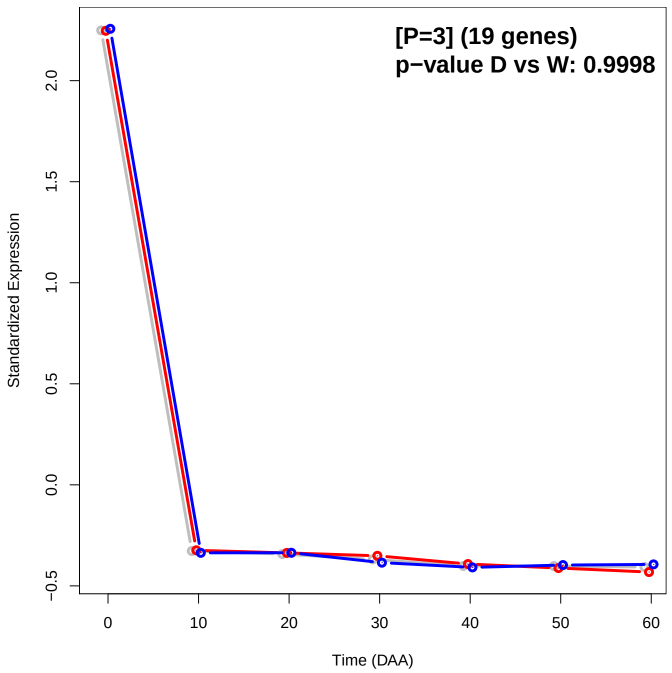

(C) Panel **(c)** in Figure 7 in main text.

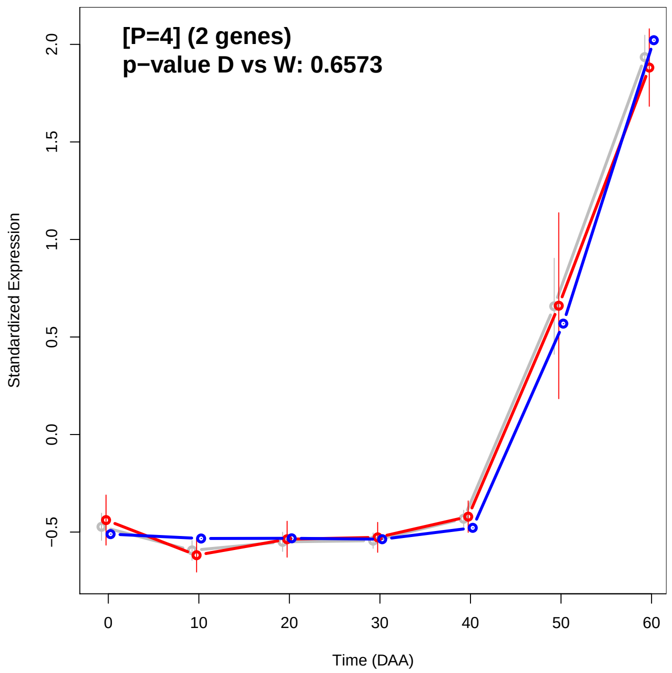

(D) Panel **(d)** in Figure 7 in main text.

FIGURE 8. Plots of average Standardized Expression Profiles (SEPs) for the 4 sets of genes that form disconnected subgraphs within the MN; Figure 7 in main text. Red lines show SEPs for Domesticated (D), blue for Wild (W) and grey for the full set of 12 genotypes. Vertical lines are 95% CI for the means at the corresponding time points. See panel (C) in Figure 7 for the plot of the 4 disconnected subgraphs. See Table 6 for gene identifiers. See column "**P**" in Table 6 for the identifiers and description of genes included in each expression pattern. Each panel includes a legend with the *p*-value for the test between the SEPs in the D and W genotypes (obtained with function "`analyze.2.SEPs()`").

In Table 5 we can see that the four disconnected graphs are very strong, by having an average of $\hat{r}$ of 0.98, and an average of q-values of 0.01, which is ten times smaller than the threshold of $Q = 0.1$ set for the *Gene2Gene* algorithm (see also Figure 3, which presents the violin plots for the distributions of $\hat{r}$ by source). In conclusion, the 4 disconnected graphs are trustworthy, because they are repeated in the whole set of 12 genotypes with an *a posteriori* error of 1×10^{-24}, calculated by taking the average of the q-values = 0.01, and also because the correlations are very strong, as shown in Table 5.

SI.3.4.1. *Pattern [**P=1**].* The network presenting the time expression pattern [**P=1**] is shown in panel (C) of Figure 7, while the expression pattern itself, i.e., the plot of the SEPs, is shown in panel (A) in Figure 8. The average SEPs of the 4 genes in the whole set of the 12 genotypes is plot in panel (A) in Figure 8 with a grey line, and resemble an "M" letter, with local maxima near to 1 standardized unit in the Y-axis, and at 10 and 30 DAA in the X-axis. However, when segregating the SEPs in the sets of domesticated (D) and wild (W) genotypes, it is notorious that the two patterns greatly differ; while the SEPs of the 6 D genotypes have the global maximum at 10 DAA, that global maximum is present later, at 30 DAA, in the 4 W genotypes. Also, the difference between D and W genotypes within pattern [**P=1**] is highly significant (p-value of the t-test $< 2.2 \times 10^{-16}$ as shown in the legend of that panel).

In fact, the 4 genes with pattern [**P=1**] are part of a set of genes with clear differences in expression patterns between D and W reported in (Martínez et al., 2021). Nonetheless, this difference was found here serendipitously when analyzing the pattern [**P=1**], and not as in Martínez et al. (2021), where the objective was to find genes with contrasting patterns between D and W. This shows how the scrutiny of particular expression patterns can lead to interesting findings with biological relevance.

It is important to fully understand that the SEP plots –as the one presented in the panel (A) of Figure 8 for the pattern [**P=1**], only summarize the average gene expression behavior. To show the concordance of gene expression patterns, Figure 9 presents an example for a single relation in a single genotype.

Figure 9 shows the SEPs of genes with identifiers 11478 and 24186, which correspond to a "kinesin-4" (XP_016552070.1) and to a Transcription Factor "high mobility group B protein 6" (XP_016555757.1), which correspond to vertices 9 and 10 in network figures (see also Table 6). Those genes, with pattern [**P=1**], appear in both, the `celcy` and `rep` networks, and the figure exemplifies its pattern in genotype "CW".

In pannel (A) of Figure 9 we see that the expression patterns of both genes are almost perfectly concordant on time in the CW genotype (the red and blue lines were slightly displaced to avoid overlapping). The almost perfect correlation ($\hat{r} = 0.9996$; $p = 6.65 \times 10^{-9}$) is shown in panel (B) of the figure by fitting a linear regression model (black line), and including the 95% confidence intervals for the regression (blue lines) as well as the 95% confidence intervals for the prediction of new observations (red lines). Given that the points corresponding to times 50, 60 and 40 are to close to each other, panel (C) of the figure gives a zoom of that part of the plot that allows to fully appreciate the small deviations from the regression model.

A crucial property of the *Gene2Gene* algorithm is that it recovers correlations between pairs of genes that are repeated in different independent sources (genotypes), even in the cases where the expression patterns of the genes differ among genotypes. To appreciate this, Figure 10 present the plot of the 4 genes in pattern **P=1** in the 6 domesticated (D) and 4 wild (W) genotypes.

In Figure 10 we can see that the general pattern in the D genotypes (pink lines) shows the average tendency seen in panel (A) of Figure 8 for D genotypes (red line in that figure), while the light blue lines resemble the average tendency of expression for W genotypes –the blue line in panel (A) of Figure 8. However, without the pair-wise selection performed by the algorithm over the times profiles per genotype, such relations will be very difficult or impossible to find, because for example some of the genes in the W genotypes present an steady state between times 20 to 30 DAA, and that behavior is consistent in all the pairs of genes within the same genotype.

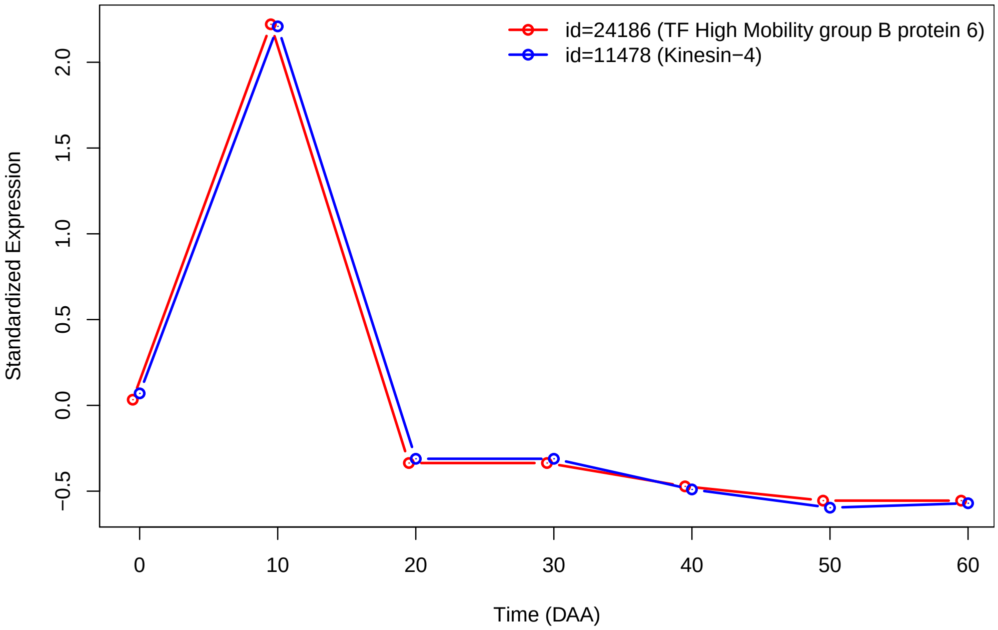

(A) Plots of the two SEPs.

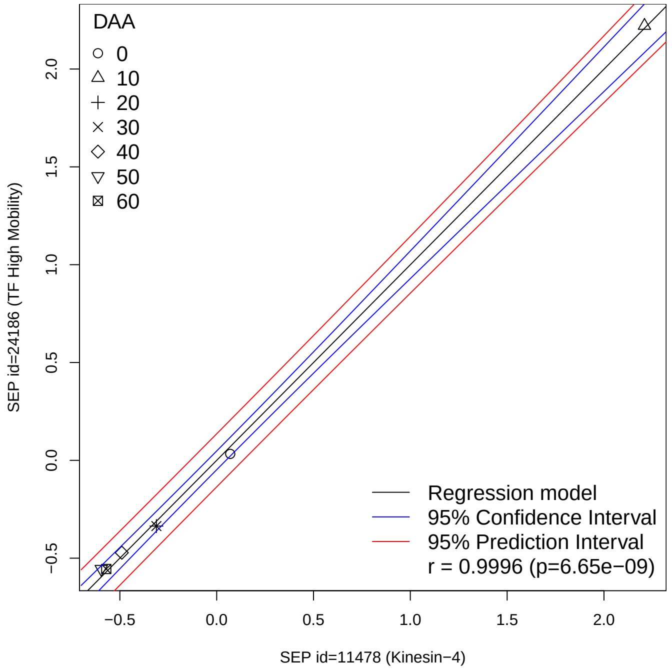

(B) Regression model SEP 1 *vs.* SEP2

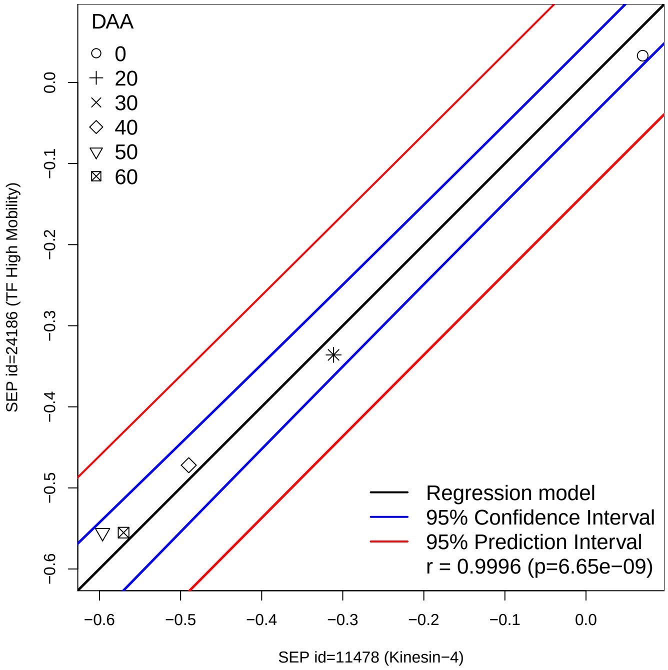

(C) Zoom of panel (B)

Figure 9. Example of concordant gene expression of two genes in the domesticated (D) genotype "CW". (A) Plots of SEPs. (B) Dot plot and regression model for the gene expression of the two genes. (C) Zoom of the bottom left hand side corner of panel (B).

The 4 genes with expression pattern **[P=1]** in panel (A) of Figure 8 are two kinesins, a microtubule associated protein and a high mobility protein (Table 6), and had been reported in *Arabidopsis* as part of cytoskeletal motors (Lee and Liu, 2004). All these four genes are annotated in the cell reproduction BP, while one of the kinesins and the high mobility protein are also annotated in the reproduction BP, and thus form part of the bridge of 6 genes that exist between the `celcy` and `rep` GFN (panels (A) and (C) in Figure 7).

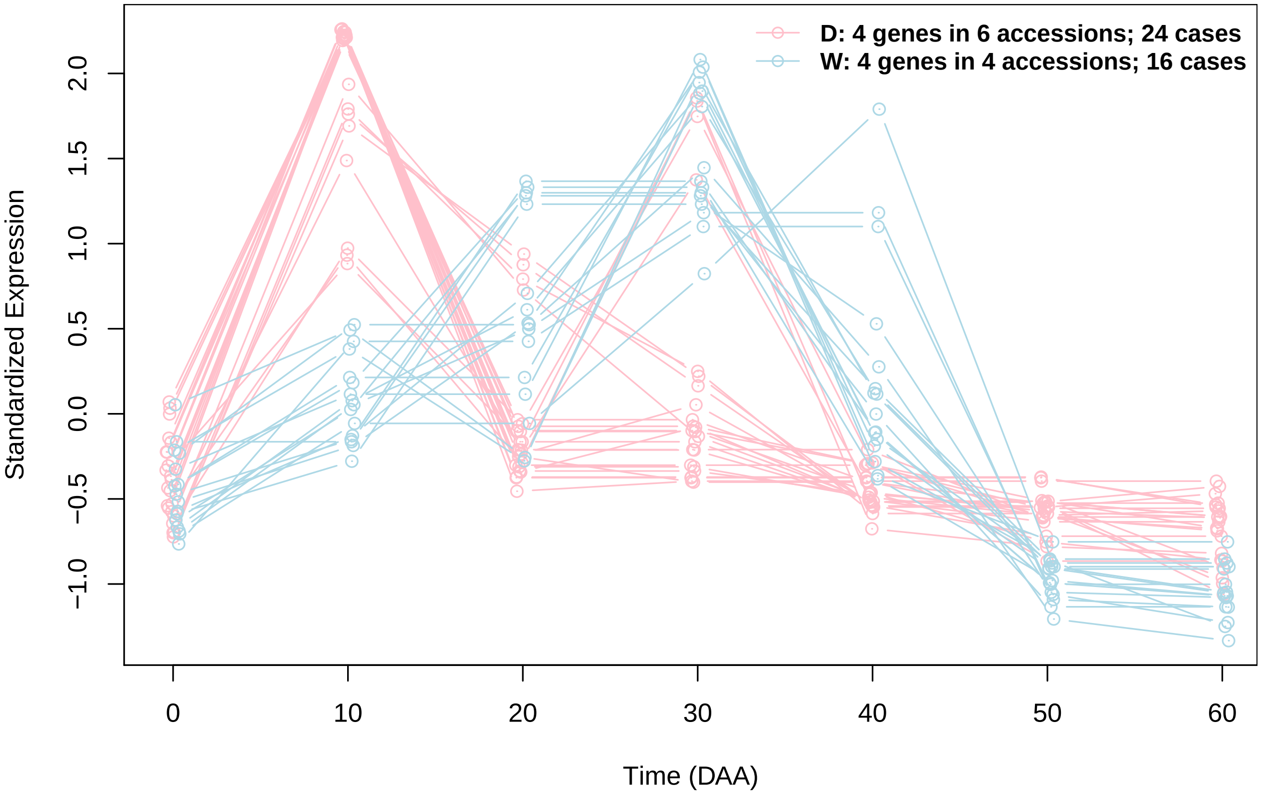

FIGURE 10. Plot of individual SEPs for genes in **P=1**.

The most interesting characteristic that present the expression patterns of the 4 genes in [**P=1**] is the asynchrony between domesticated (D) and wild (W) genotypes; while in the 6 D genotypes the maximum expression is early, at 10 DAA, in the 4 W genotypes that maximum is at 30 DAA. Those 4 genes are included in the set of 524 genes previously reported by us in (Martínez et al., 2021) as relevant for the chili pepper domestication process; furthermore, the kinesin 3, with pattern [**P=2**], is included in the asynchronous gene co-expression network in that publication.

Of particular interest within the genes with expression pattern [**P=1**] is the "high mobility group B protein 6" (gene vertex 10 in the figures, protein XP_016555757.1; see Table 6), which is a TF of the family that has been reported to have a role in stress response in *Arabidopsis* (Kwak et al., 2007), and our results here show that very likely the expression of this gene has been modified by the domestication process.

SI.3.4.2. *Pattern [**P=2**].* Panel (B) in Figure 8 shows the average SEPs for the 10 genes included in the subgraph [**P=2**]. The general pattern in the 12 genotypes (grey line) is characterized by having a global maximum at 10 DAA which generally decline to reach the global minimum at the mature fruit state at 60 DAA. Even when there is a significant difference ($p < 2.2 \times 10^{-16}$) between the average SEPs in the D and W genotypes, the differences are less dramatic than the ones observed for pattern [**P=1**], being larger only at 20, 30, 50 and 60 DAA.

The larger and most remarkable difference between D and W genotypes in pattern [**P=2**] happens at 30 DAA. Furthermore, the tendency of average expression from 20 to 30 DAA is to increase in D, while it is to decrease in W genotypes. This is on line with the fact that in [**P=1**] we have an asynchronicity in the maxima between D and W; in [**P=2**] at least some of the genes in W show a local maximum at 30 DAA. Figure 11 presents the plot of the individual SEPs of all cases for genes with expression pattern **P=2**.

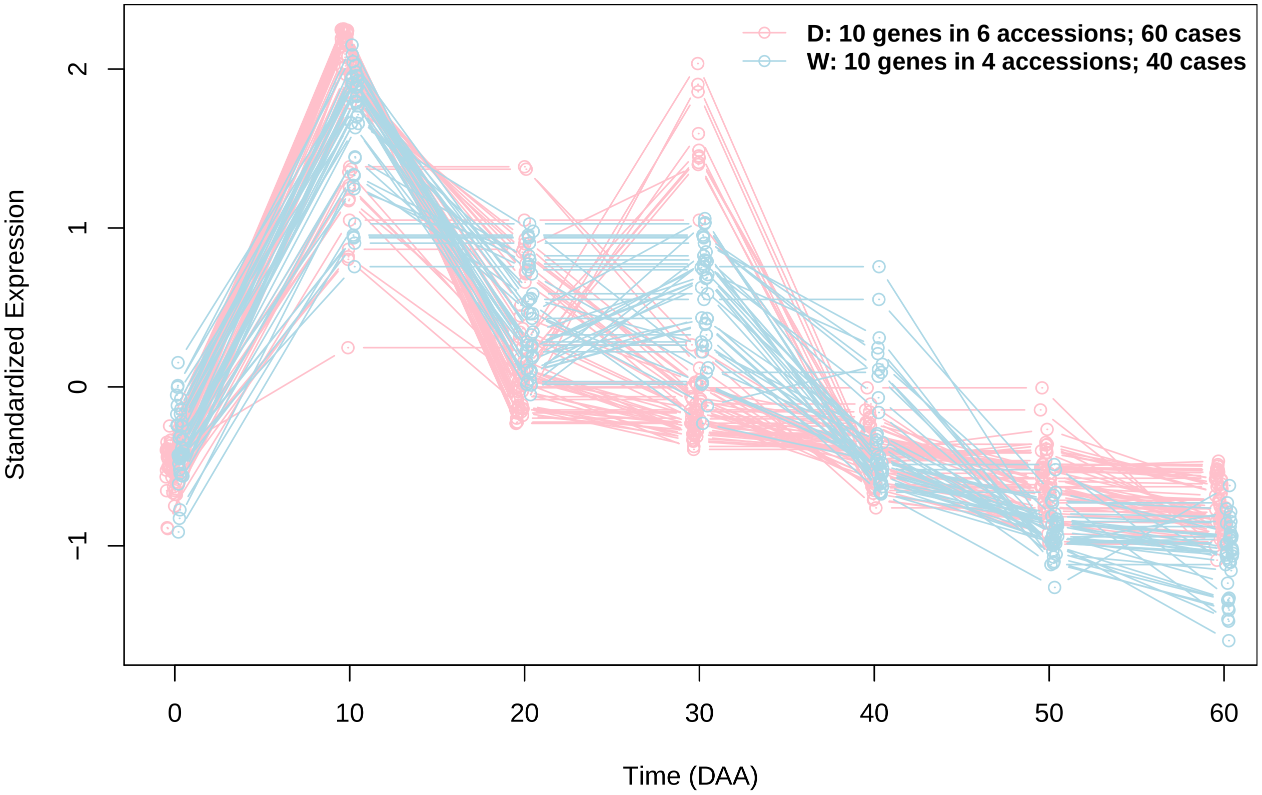

FIGURE 11. Plot of individual SEPs for genes in **P=2**.

In Figure 11 we have plots of SEPs for 10 genes in the 6 D genotypes, i.e., a total of 60 SEPs plot as pink lines, while the 10 genes in the 4 W genotypes are shown as the 40 light blue lines. In that figure we can see that some D and W cases present local maxima at 30 DAA, but that happens in fewer D than W cases. Furthermore, many W cases show a steady tendency in the time period from 10 to 30 or even 40 DAA, which is less common in D cases.

As for pattern **P=1**, in the case of pattern **P=2** we can appreciate the fact that the *Gene2Gene* algorithm recovers the pairwise correlation that happens between the same genes, even when these genes present heterogeneity of gene expression in different genotypes. In other words, when two different genes have highly consistent time expression patterns in all genotypes, the algorithm will recover them into a GFN, even if the expression patterns differ between genotypes.

The 10 genes with expression pattern [**P=2**] are annotated in the `celcy` GFN (panel (C) in Figure 7) and have an expression pattern which global maximum is early in fruit development at 10 DAA (panel (A) of Figure 8). Even when there are significant differences (p-value $< 2.2 \times 10^{-16}$) between D and W, those differences happen in later times, mainly at 30 and 60 DAA, and will not be further examined.

The group of 10 genes with expression pattern [**P=2**] is mostly formed (7 of 10) by paralog genes coding for histone H3.2 proteins (Table 6). Histone H3 proteins are reported to play fundamental roles in DNA packaging, gene transcription, and the transmission of epigenetic states (Hödl and Basler, 2012), and thus it results congruent that the point of maximum transcription of this group (at 10 DAA) coincides with the maximum rate of fruit development. Interestingly, the maximum expression pattern of histone H3.2 genes does not appears to be modified by the domestication process, been identical in all 12 genotype studied, and this is in contrast with other cell cycle genes, which presented a difference of maximum only in wild genotypes (see panel (A); [**P=1**] in Figure 8).

The TF candidates to be regulating the genes with expression pattern [**P=2**] produced a highly connected network (Figure 13), and within the TF candidates found are three forms of histone H2A (Table 7), which

family had been reported to be involved in transcriptional control in *Arabidopsis* (Redon et al., 2002) and had also been reported in relation to resistance to geminivirus in *Capsicum* (Góngora-Castillo et al., 2012).

It is intriguing that only histones H3 and H2A present the highly concordant expression pattern [**P=2**] in all 12 genotypes sampled. This could be due to the fact that the other histones that form the nucleosome (H4 and H2B) have an expression pattern that differs among genotypes.

SI.3.4.3. *Pattern [**P=3**].* Panel (C) in Figure 8 shows the average SEPs for the 19 genes included in the subgraph [**P=3**]. In contrast with the cases in panels (A) and (B) in that figure, which correspond to patterns [**P=1**] and [**P=2**], the gene expression pattern in [**P=3**] is highly homogeneous among genotypes; in fact, there are no significant differences between the D and W groups (p-value = 0.9998).

The expression pattern [**P=3**] resembles the letter "L"; i.e., it is high at the mature flower at 0 DAA, then sharply decreases from 0 to 10 DAA and then stays in a relatively steady state from 10 DAA up to the mature fruit at 60 DAA. For this we can call the set of genes that present such pattern "preferentially expressed in flower" (PEF), adding that they are lowly and steadily expressed during all fruit development.

In fact, PEF genes are the most frequent among all genes detected in our data. Of the 22374 genes consistently expressed in all 12 genotypes, 4379 ($\approx 20\%$) present a fully steady state during all the time sampled (0 to 60 DAA) in at least some of the genotypes, but a total of 7497 ($\approx 33.5\%$) shows the PEF pattern by having a maximum standardized expression ≥ 2 at 0 DAA in one or more genotypes.

The shift from high expression at 0 DAA to very low or null expression in all subsequents stages in the fruit that present PEF genes, signals the dramatic tanscriptome transformation between two very different organs: flower and fruit. Thus, it is remarkable but not surprising, that the largest number of genes (19) in all 4 disconnected graphs forming the MN belong to this category of PEF genes, presenting the [**P=3**] expression pattern and including genes within the three GFN, `calcy`, `rep` and `vir`, that integrate the MN. Gene distribution by GFN in this pattern is: 13 in `celcy` of which 8 are exclusive to that GFN, 5 in `rep` of which 4 are exclusive of that GFN, and 4 in `vir`, of which 2 are exclusive of that GFN (see panel (C) in Figure 7).

The gene with more edges (direct connections) of the 19 in the PEF network ($v_i = 25$ in Table 6), is identified as a "*BTB/POZ domain-containing protein DOT3 isoform X2*". In general DOT genes are associated with leaf development (Petricka et al., 2008), and in particular DOT3 has been reported as responsive to light conditions in leaves of *Camellia oleifera* (Song et al., 2020). The fact that in our data DOT3 is highly expressed in the mature flower at 0 DAA, but has a very low or null expression during all fruit development, suggest that this gene could play a role in floral structures which are absent in the fruit, as for example petals and sepals.

Of the 19 genes in the PEF network, 7 ($\approx 37\%$) are TF (Table 6). The TF with $v_i = 15$ in Table 6 can be considered as a regulator hub of the network, by being the one with more connections (see panel (C) in Figure 7) and also by forming part of both, the `celcy` and the `rep` GFN. This gene codes for a protein identified as "*homeobox-leucine zipper protein ATHB-52*". The product of this gene "Q9FN29" or "*ATB52_ARATH*" in *Arabidopsis*, is annotated in UniProt in the GO BP as "*positive regulation of transcription, DNA-templated*", but also and interestingly as "*response to absence of light*" and "*response to blue light*" with TAIR as source. In potato –a Solanaceae as *Capsicum*, the ortholog of ATHB-52 has been reported as differentially regulated in response to a virus infection (Jeevalatha et al., 2017). As the previously commented gene *DOT3*, this TF could be also involved in the development of flower specific structures, given that is lowly or non expressed during fruit development.

Of the other 6 TF in the [**P=3**] expression pattern, 4 are highly connected within the `celcy` GFN. These, with vertices 13, 16, 18 and 20 in panel (C) of Figure 8, are identified as a "*homeobox-leucine zipper protein HAT5*", "*floral homeotic protein PMADS 2*", "*agamous-like MADS-box protein AGL19*"

and "*homeobox protein knotted-1-like 1*, respectively, while the ones with vertices 33 ("*transcription factor FAMA-like*") and 34 ("*transcription factor PRE1-like*") are only in the `rep` and `vir` GFN, respectively (Table 6).

In summary, the genes with expression pattern [**P=3**] –a subset of the PEF set, are highly expressed at the mature flower but lowly expressed during fruit development, and form a connected and highly homogeneous part of the Meta Network (MN), which is rich in TF and groups genes that are likely to be important mainly in the flower.

SI.3.4.4. *Pattern [**P=4**].* Pattern [**P=4**], presented in panel (D) of Figure 8 is the less populated of the 4 patterns, being present in only a pair of genes. As in the case of PEF genes (pattern [**P=3**]) there are not significant differences between D and W (p-value = 0.6573), and the [**P=4**] pattern is almost inverse to the [**P=3**] one, showing a inverted "L" shape.

In fact, [**P=4**] is characterized for an almost steady state from 0 up to 40 DAA, increasing from 40 to 50 and 50 to 60 DAA, and thus this pattern is typical of genes that are very active during fruit maturation. Genes with pattern [**P=4**] are also frequent in the whole data set; a total of 5927 genes present such pattern in at least one genotype, representing approximately 26% of the total.

The expression pattern [**P=4**] in panel (D) of Figure 8 is followed by 2 genes of the `celcy` GFN (panel (C) in Figure 7). As mentioned above, those genes are at low expression from 0 to 40 DAA, but from that point their expression increases linearly up to full maturity at 60 DAA, without differences between domesticated and wild genotypes. These two genes correspond to a "`uridine-cytidine kinase C`" and to the TF "`LOB domain-containing protein 1`", with vertices labeled with numbers 11 and 12 in the figures, respectively (Table 6).

Uridine-cytidine kinases are generally involved in the pyrimidine salvage pathway (Chen and Thelen, 2011), and in *Arabidopsis* had been related to chloroplast biogenesis (Ohler et al., 2019), while in maize its differential expression has been linked with salt-stress tolerance (Zhao et al., 2019). Concordantly, in Portulacineae the evolution of this gene family had been linked with the adaptation to extreme and harsh environments (Wang et al., 2019).

On the other hand, the LOB gene family encodes plant-specific transcription factors which play important roles in plant growth and development (Zhang et al., 2020), and in particular, the gene coding for the LOB domain-containing protein 1 has been related to the mechanism of litchi fruit senescence (Zhang et al., 2021).

In chili pepper the two genes with expression pattern [**P=4**] could be involved in the chromoplast biogenesis from chloroplast –because the expression pattern strongly increases from 40 to 60 DAA, coinciding with chromoplast biogenesis and carotenoid biosynthesis (Gómez-García and Ochoa-Alejo, 2013).

SI.3.5. **Finding Transcription Factors (TF) consistent with expression pattern [P=2].** The only subgraph within the MN that does not include TF is the one with pattern [**P=2**] (panel (B) in Figure 8). That subgraph can be seen in panel (C) in Figure 7, and is constituted by 10 genes, all belonging to the `celcy` GFN, none of which is a TF (see Table 6 for the identity of such genes).

The *Gene2TF* algorithm, implemented in the "*Salsa*" function "`g2g.TFcandidates`", allows to find TF that are good candidates to be regulating an Structural Gene (SG) by having highly correlated expression patterns with a gene of interest. As in the *Gene2Gene* algorithm, in the case of the *Gene2TF* the main strength of the method is to find a strong correlation repeated in various independent data sources (genotypes).

We run the "`g2g.TFcandidates`" for each one of the 10 genes with pattern [**P=2**] with the same levels of stringency that we used in for the *Gene2Gene* algorithm, and asking that the relation was found in every one of the 12 genotypes. Details of the calculations in R are in the "`# * find TF candidates`" section of the Appendix SI.5.1.

Briefly, the "`g2g.TFcandidates`" was run for each one of the 10 SG with pattern [**P=2**], and the identifiers of the TF candidates that resulted were tabulated. We took into account only the TF candidates that were found repeatedly for all 10 SG and in all 12 genotypes. This procedure produced a set of 10 TF candidates. Next, we run the *Gene2Gene* algorithm including the 20 genes, i.e., the 10 SG with pattern [**P=2**] plus the 10 TF candidates selected. The resulting network included all 20 genes, linked by a total of 77 relations, and it is presented in Figure 12. We also corroborated the strength and significance of all the 77 pair correlations, observing that the average value of $\hat{r}$ was 0.9767, and the tests performed gave an average value of q of 7.5×10^{-4}, which implies a vanishingly small error probability of approximately 3×10^{-38}, which implies a very strong network which is highly likely to be shown causal links between the genes.

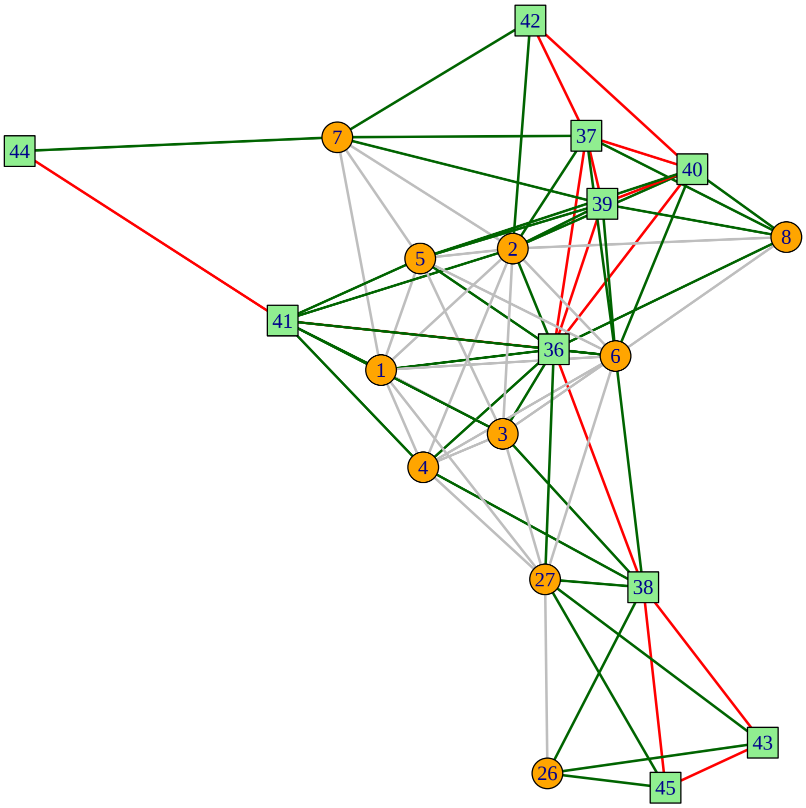

Figure 12. Plot of the network formed by the Structural Genes (SG) with pattern "[**P=2**]" (orange circles) after adding Transcription Factors (TF) candidates (green squares). Relations colors: between SG in grey, between SG and TF in green and between TF in red. Vertices definitions for SG in Table 6, and for TF in Table 7.

Figure 12 shows the network of SG with pattern "[**P=2**]" (orange circles), which also include the 10 TF candidates found (green squares). Each gene is labeled with its vertex identifier (`vi`); the identity of SG can be consulted in Table 6, while the identity of the TF is presented in Table 7. As said above, this network comprises 20 genes and 77 gene pair relations, and the number of connections (edges) vary between 2 and 13 with a median of 8 connections per gene.

In Figure 13 we can see that the average of SEPs for the 10 SG and the 10 TF are highly concordant. The average standardized expression of the genes begins at the mature flower state at approximately -0.5, but reach their global maximum of almost 2 standardized units at 10 DAA –when the activity of mitosis is known to be larger in chili pepper (Martínez et al., 2021). From 10 to 20 DAA the average standardized

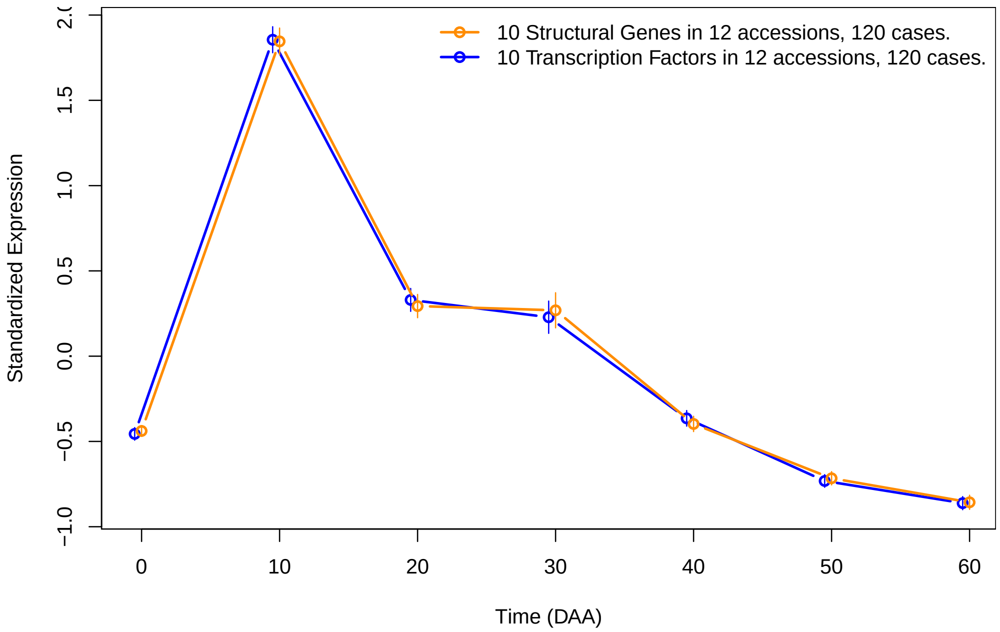

FIGURE 13. Average SEPs for Structural Genes (SG) with pattern "**[P=2]**" (in orange) and Transcription Factors (TF) candidates (in blue). See Figure 12 for the network.

expression of all genes rapidly decrease, stays in an almost steady state from 20 to 30 DAA and then decrease almost linearly to reach their global minimum at the mature fruit at 60 DAA.

In conclusion, when no TF appear in a network, the *Gene2TF* can be applied to find one or more candidates to be regulating the genes of interest. Even when such TF candidates are selected only from bioinformatic procedures, and will need experimental corroboration for other means, the procedure shorten the list from (potentially) all the TF annotated in the *Capsicum* genome, up to a few ones.

SI.4. Concluding remarks

SI.4.1. **Generality and limitations of the *Gene2Gene* algorithm.** The main limitation for the application of the *Gene2Gene* algorithm is the need of time expression profiles for the phenomenon of interest from independent genotypes. Time course gene expression experiments are frequently employed, and the methods to analyze them are increasing in accuracy and sophistication (Spies and Ciaudo, 2015; Spies et al., 2019). Gene expression and regulation is intrinsically dynamic, as transcription generally changes during the different stages of cell differentiation and across cell types (Strober et al., 2019).

There are already many methods to obtain networks from co-expression data (López-Kleine et al., 2013), which allow genome wide estimation of gene relations, as for example ARACNE (Margolin et al., 2006; Lachmann et al., 2016), with versions that can take into account time changes (Zoppoli et al., 2010). Even so, none of these methods takes into account the fact that finding gene relations from independent genotypes allows to filter relations that could exist in single or a few genotypes from those that are consistently repeated in many or all cases.

A unique characteristic of the *Gene2Gene* algorithm is that it measures the robustness of a gene relation by a formula that takes into account not only the size and significance of the correlation, but critically, the number of times that such relation is found on independent data sources (see section SI.2.2 in Methods). This approach allows to judge if a relation between genes is present in general or limited to particular genotypes. In turn, this implies the possibility to reach a better understanding of the hierarchical nature of gene relations, that could be present only in subsets of genotypes.

SI.4.2. **Constructing GFN: a bottom-up approach.** In the results presented to exemplify our method, we began by estimating three GFN for BP cell cycle "`celcy`", reproduction "`rep`" and response to virus "`vir`" (Figure 6). Given that these three GFN share genes, we combined them into a MN (Figure 7). The MN contained four disconnected subgraphs, and that implies that we have four highly contrasting expression profiles, which are shown in Figure 8. Finally, after observing that one of the four disconnected subgraphs –the one with pattern **[P=2]** in panel B of Figure 8, did not contained TF, we searched for TF candidates that could be regulating one or more of the genes included in that disconnected subgraph (Figure 12).

The first interesting fact that we uncover is that genes, even when annotated into a single BP, can present highly divergent time expression patterns that lead to disconnected networks. That was evident for the `celcy` and `rep` GFN, while in the `vir` all 4 genes share exactly the same gene expression profile and form a fully connected network. From this we can conclude that gene annotation in the same BP does not implies uniformity of time expression profile, and that interpretation is likely to be more fruitful if focus on subgraphs that share a highly concordant time expression profile, as the four cases found in the MN.

SI.5. Appendices

Table 6. Identification of genes included into figures.

v_i	`id`	**P**	GFN	Protein	Description	TF?
1	10543	**2**	C	XP_016559802.1	histone H3.2	No
2	11233	**2**	C	XP_016539307.1	histone H3.2	No
3	15420	**2**	C	XP_016559802.1	histone H3.2	No
4	16355	**2**	C	XP_016539307.1	histone H3.2	No
5	27030	**2**	C	XP_016559802.1	histone H3.2	No
6	27031	**2**	C	XP_016554909.1	histone H3.2	No
7	24773	**2**	C	XP_016547668.1	interactor of constitutive active ROPs 3 isoform X1	No
8	33587	**2**	C	XP_016572466.1	TIMELESS-interacting protein	No
9	11478	**1**	$C \cap R$	XP_016552070.1	kinesin-4	No
10	24186	**1**	$C \cap R$	XP_016555757.1	high mobility group B protein 6	Yes
11	11680	**4**	C	XP_016553276.1	uridine-cytidine kinase C	No
12	24794	**4**	C	XP_016548470.1	LOB domain-containing protein 1	Yes
13	15639	**3**	C	XP_016548225.1	homeobox-leucine zipper protein HAT5	Yes
14	23951	**3**	$C \cap R$	XP_016572058.1	kinase-interacting protein 1	No
15	24093	**3**	$C \cap R$	XP_016567874.1	homeobox-leucine zipper protein ATHB-52	Yes
16	24172	**3**	C	XP_016575228.1	floral homeotic protein PMADS 2	Yes
17	34575	**3**	C	XP_016541401.1	clathrin coat assembly protein AP180	No
18	35535	**3**	C	XP_016567907.1	agamous MADS-box protein AGL19	Yes
19	35870	**3**	C	XP_016563542.1	probable myosin-binding protein 5 isoform X1	No
20	1612	**3**	C	XP_016538789.1	homeobox protein knotted-1 1	Yes
21	18785	**3**	C	XP_016581620.1	uncharacterized protein LOC107878997	No
22	4788	**3**	$C \cap V$	XP_016538722.1	kinesin-4	No
23	6576	**3**	$C \cap R$	XP_016572104.1	glucan endo-1,3-beta-glucosidase 8 isoform X1	No
24	34420	**3**	C	XP_016580671.1	cyclin-U1-1	No
25	8415	**3**	$C \cap R \cap V$	XP_016573212.1	BTB/POZ domain-containing protein DOT3 isoform X2	No
26	6072	**2**	C	XP_016538301.1	65-kDa microtubule-associated protein 5	No
27	6967	**2**	C	XP_016559802.1	histone H3.2	No
28	673	**1**	C	XP_016564755.1	65-kDa microtubule-associated protein 3	No
29	15446	**1**	C	XP_016541615.1	kinesin-3 isoform X3	No
30	12729	**3**	R	XP_016552286.1	G-type lectin S-receptor serine/threonine-protein kinase RLK1	No
31	19646	**3**	R	XP_016576684.1	G-type lectin S-receptor serine/threonine-protein kinase RLK1	No
32	25868	**3**	R	XP_016567423.1	subtilisin protease SBT3.6	No
33	30576	**3**	R	XP_016574783.1	transcription factor FAMA	Yes
34	13178	**3**	V	XP_016561417.1	transcription factor PRE1	Yes
35	33679	**3**	V	XP_016577636.1	calcium-dependent protein kinase 17	No

Column descriptions:

v_i – Number of node (gene) in all figures.
`id` – Numeric identifier of the gene in *Salsa*.
P – Expression type identifier; number of disconnected subgraph; see annotations "[**P=**]" in panel (C) of Figure 7 and expression patterns in Figure 8.
GFN – In which of the GFN appear the node:
C = Cell Cycle (`celcy`), R = Reproduction (`rep`), V = Response to Virus (`vir`).
Protein – Protein identifier (link to the NCBI). Description – Protein short description.
TF? – Is the gene annotated as a Transcription Factor? (Yes or No).

Table 7. Identification of Transcription Factor (TF) candidates included in Figure 12.

v_i	id	Protein	Description
36	10038	XP_016538629.1	histone H2A.1-like
37	20830	XP_016574575.1	high mobility group B protein 7-like
38	21120	XP_016547748.1	E3 ubiquitin-protein ligase ORTHRUS 2-like
39	25590	XP_016545608.1	transcriptional regulator ATRX homolog
40	30554	XP_016548500.1	histone H2A-like
41	30563	XP_016556665.1	histone H2A
42	30900	XP_016566744.1	trihelix transcription factor GTL1-like
43	18607	XP_016560606.1	ATP-dependent DNA helicase DDM1
44	20355	XP_016562806.1	transcription factor TCP14
45	3529	XP_016554726.1	auxilin-like protein 1

SI.5.1. **R code.** The box below we present the main operations performed using data and functions in the R package "*Salsa*" (v 1.0) (Martínez and Escoto-Sandoval, 2021) to obtain the results of this research. You could reproduce the results and examine details of the objects created by running in order each one of the R statements that appear after the prompt, i.e., after ">".

All figures of networks were obtained using the R package "*igraph*" (Csardi and Nepusz, 2006), however, we do not present here the R code to obtain such figures. Subsections of this appendix are shown in the main text as for example "Appendix SI.5.1 `*celcy`", which you can find within the box below. Details of the object created can be obtained examining their components. See the help facility of each one of the functions within the "*Salsa*" package. All objects obtained within this section are available to be downloaded from the link "MainResults.RData".

```
-----------------------------------------------------------------------------------------
##### First steps
# Instal the Salsa package.
# and load it into your environment:
> library(Salsa)

# Please, examine the help of the package and main functions;
# at least review
> ? Salsa
> ? core.g2g
> ? g2g

##### *celcy (Cell cycle)
# Obtain the ids of genes annotated in "cell cycle"
# See to which GO identifier the BP corresponds.
> all.GO[all.GO$GO.desc=="cell cycle",]
# Isolate the ids
> celcy.ids <- GO.annot$id[GO.annot$GO=="GO:0007049"]
# celcy.ids contains 471 gene identifiers, however not all those
# genes are expressed in all genotypes during fruit development.
# We need only those in our vector:
> celcy.ids <- intersect(celcy.ids, get.SEP(ExistInAll=TRUE)$id)
# See how many of those genes you are going to study
> length(celcy.ids)
# Obtain the "core" object (with all stuff needed later)
# WARNING: Next line took approximately 4 hours in my laptop!
```

```
> celcy.core <- core.g2g(ids=celcy.ids)
# Obtains the object with the GFN for "celcy"
> celcy.g2g.12 <- g2g(celcy.core, min.fdr=0.1, min.r2=0.7, n.min.acc=12)
# Use function analyze.g2g to give a summary of the results in celcy.g2g.12
> analyze.g2g(celcy.g2g.12)

Result of running function "g2g" with a set of 352 genes
(ids in the input) and with main parameters:
                               in.acc min.fdr min.r2 m.a.quan n.min.acc
1 AS,CM,CO,CQ,CW,JE,QC,QU,SR,ST,SY,ZU     0.1    0.7     0.05        12

There were 81 pairs of genes which surpassed the main parameters in at least 12 genotypes.
Those 81 pairs are formed by a total of 29 different genes.
Thus a total of 323 genes (91.76%) do not appear in the output.
The method estimated relations between 8.239% of the genes in the input.

Number of times that each gene id (upper row) participates into winner pairs (bottom row)
[Useful to detect "hub" genes].

Table of gene ids in winners.
 4788  6576 15639 23951 24093 24172 34575 35535 35870  1612  8415 11233
   10    10    10    10    10    10    10    10    10     9     9     7
10543 15420 27031  6967 16355 27030   673 11478 24186  6072 11680 15446
    6     6     6     5     5     4     3     2     2     1     1     1
18785 24773 24794 33587 34420
    1     1     1     1     1
Same table than above in rounded percentages:
Table of gene ids in winners.
 4788  6576 15639 23951 24093 24172 34575 35535 35870  1612  8415 11233
    6     6     6     6     6     6     6     6     6     6     6     4
10543 15420 27031  6967 16355 27030   673 11478 24186  6072 11680 15446
    4     4     4     3     3     2     2     1     1     1     1     1
18785 24773 24794 33587 34420
    1     1     1     1     1

Table of genotypes that are included in winners.
AS CM CO CQ CW JE QC QU SR ST SY ZU
81 81 81 81 81 81 81 81 81 81 81 81
Same table than above in rounded percentages:
Table of genotypes that are included in winners.
AS CM CO CQ CW JE QC QU SR ST SY ZU
 8  8  8  8  8  8  8  8  8  8  8  8

A total of 12 of the 12 genotypes (100%) are found in at least one winner.

Summary for the number of genotypes in each winner
   Min. 1st Qu.  Median    Mean 3rd Qu.    Max.
     12      12      12      12      12      12

Table of logical variable "r.pos"
TRUE
  81
```

```
Summary of average correlation coefficient by logical variable "r.pos".$‘TRUE‘
   Min. 1st Qu.  Median    Mean 3rd Qu.    Max.
 0.9552  0.9798  0.9854  0.9859  0.9946  1.0000

Summary of average Q values (for FDR) in all winners.
    Min.  1st Qu.   Median     Mean  3rd Qu.     Max.
0.000000 0.001032 0.006737 0.007290 0.010056 0.034503

Summary of average m.a (maximum absolute) by logical variable "r.pos".
$‘TRUE‘
    Min.  1st Qu.   Median     Mean  3rd Qu.     Max.
0.005129 0.145449 0.251039 0.232570 0.315111 0.461547

##### *rep (Reproduction)
# Obtain the ids of genes annotated in "reproduction"
# See to which GO identifier the BP corresponds.
> all.GO[all.GO$GO.desc=="reproduction",]
# Isolate the ids
> rep.ids <- GO.annot$id[GO.annot$GO=="GO:0000003"]
# rep.ids contains 304 gene identifiers, however not all those
# genes are expressed in all genotypes during fruit development.
# We need only those in our vector:
> rep.ids <- intersect(rep.ids, get.SEP(ExistInAll=T)$id)
# This operation let us with 228 identifiers.
# Obtain the "core" object (with all stuff needed later)
# WARNING: Next line took approximately 3/4 of an hour in my laptop!
> rep.core <- core.g2g(rep.ids)
# Obtains the object with the GFN for "celcy"
> rep.g2g.12 <- g2g(rep.core, min.fdr=0.1, min.r2=0.7, n.min.acc=12)
# Use function analyze.g2g to give a summary of the results in rep.g2g.12
> analyze.g2g(rep.g2g.12)

Result of running function "g2g" with a set of 228 genes (ids in the input)
and with main parameters:
  in.acc min.fdr min.r2 m.a.quan n.min.acc
1            0.1    0.7     0.05        12

There were 29 pairs of genes which surpassed the main parameters in at least 12 genotypes.
Those 29 pairs are formed by a total of 10 different genes.
Thus a total of 218 genes (95.61%) do not appear in the output.
The method estimated relations between 4.386% of the genes in the input.

Number of times that each gene id (upper row) participates into winner pairs (bottom row)
[Useful to detect "hub" genes].

Table of gene ids in winners.
 6576  8415 12729 19646 23951 24093 25868 30576 11478 24186
    7     7     7     7     7     7     7     7     1     1
Same table than above in rounded percentages:
Table of gene ids in winners.
 6576  8415 12729 19646 23951 24093 25868 30576 11478 24186
```

```
   12    12    12    12    12    12    12    12     2     2

Table of genotypes that are included in winners.
AS CM CO CQ CW JE QC QU SR ST SY ZU
29 29 29 29 29 29 29 29 29 29 29 29
Same table than above in rounded percentages:
Table of genotypes that are included in winners.
AS CM CO CQ CW JE QC QU SR ST SY ZU
 8  8  8  8  8  8  8  8  8  8  8  8

A total of 12 of the 12 genotypes (100%) are found in at least one winner.

Summary for the number of genotypes in each winner
   Min. 1st Qu.  Median    Mean 3rd Qu.    Max.
     12      12      12      12      12      12

Table of logical variable "r.pos"
TRUE
  29

Summary of average correlation coefficient by logical variable "r.pos".$‘TRUE‘
   Min. 1st Qu.  Median    Mean 3rd Qu.    Max.
 0.9814  0.9880  0.9948  0.9934  0.9966  1.0000

Summary of average Q values (for FDR) in all winners.
    Min.  1st Qu.   Median     Mean  3rd Qu.     Max.
0.000000 0.001023 0.001214 0.002574 0.003859 0.012522

Summary of average m.a (maximum absolute) by logical variable "r.pos".
$‘TRUE‘
    Min.  1st Qu.   Median     Mean  3rd Qu.     Max.
0.005129 0.090602 0.139788 0.150053 0.269625 0.323941

##### *vir (Response to virus)
# Obtain the ids of genes annotated in "response to virus"
# See to which GO identifier the BP corresponds.
> all.GO[all.GO$GO.desc=="response to virus",]
# Isolate the ids
> vir.ids <- GO.annot$id[GO.annot$GO=="GO:0009615"]
# vir.ids contains 53 gene identifiers, however not all those
# genes are expressed in all genotypes during fruit development.
# We need only those in our vector:
> vir.ids <- intersect(vir.ids, get.SEP(ExistInAll=TRUE)$id)
# This operation let us with 33 identifiers.
# Obtain the "core" object (with all stuff needed later)
> vir.core <- core.g2g(ids=vir.ids)
# Obtains the object with the GFN for "vir"
> vir.g2g.12 <- g2g(vir.core, min.fdr=0.1, min.r2=0.7, n.min.acc=12)
# Use function analyze.g2g to give a summary of the results in vir.g2g.12
> analyze.g2g(vir.g2g.12)

Result of running function "g2g" with a set of 33 genes (ids in the input)
```

```
and with main parameters:
                               in.acc min.fdr min.r2 m.a.quan n.min.acc
1 AS,CM,CO,CQ,CW,JE,QC,QU,SR,ST,SY,ZU     0.1    0.7     0.05        12

There were 6 pairs of genes which surpassed the main parameters in at least 12 genotypes.
Those 6 pairs are formed by a total of 4 different genes.
Thus a total of 29 genes (87.88%) do not appear in the output.
The method estimated relations between 12.12% of the genes in the input.

Number of times that each gene id (upper row) participates into winner pairs (bottom row)
[Useful to detect "hub" genes].

Table of gene ids in winners.
 4788  8415 13178 33679
    3     3     3     3
Same table than above in rounded percentages:
Table of gene ids in winners.
 4788  8415 13178 33679
   25    25    25    25

Table of genotypes that are included in winners.
AS CM CO CQ CW JE QC QU SR ST SY ZU
 6  6  6  6  6  6  6  6  6  6  6  6
Same table than above in rounded percentages:
Table of genotypes that are included in winners.
AS CM CO CQ CW JE QC QU SR ST SY ZU
 8  8  8  8  8  8  8  8  8  8  8  8

A total of 12 of the 12 genotypes (100%) are found in at least one winner.

Summary for the number of genotypes in each winner
   Min. 1st Qu.  Median    Mean 3rd Qu.    Max.
     12      12      12      12      12      12

Table of logical variable "r.pos"
TRUE
   6

Summary of average correlation coefficient by logical variable "r.pos".$`TRUE`
   Min. 1st Qu.  Median    Mean 3rd Qu.    Max.
 0.9869  0.9876  0.9944  0.9936  0.9991  0.9999

Summary of average Q values (for FDR) in all winners.
     Min.   1st Qu.    Median      Mean   3rd Qu.      Max.
2.781e-07 1.461e-05 9.510e-04 1.256e-03 2.563e-03 2.833e-03

Summary of average m.a (maximum absolute) by logical variable "r.pos".
$`TRUE`
   Min. 1st Qu.  Median    Mean 3rd Qu.    Max.
0.01392 0.05658 0.15444 0.16030 0.28120 0.29160

##### *MN (meta network)
```

```
# The meta network is defined by concatenating the matrices
# rows of the "celcy", "rep" and "vir" objects
# (which resulted from previous operations)
# Note that the next R statement expands in three lines.
> met.mat <- rbind(celcy.g2g.12$results.gn$mat4graph,
 rep.g2g.12$results.gn$mat4graph,
 vir.g2g.12$results.gn$mat4graph)
# Note: met.mat was used to plot the MN (meta network).

 ##### OPTIONAL
 # Make a list with the objects created and save it:
 > MainResults <- c(
"celcy.ids", "celcy.core", "celcy.g2g.12",
"rep.ids", "rep.core", "rep.g2g.12",
"vir.ids", "vir.core", "vir.g2g.12",
"met.mat")
> save(list=MainResults, file="MainResults.RData")

##### * find TF candidates
# Note: Identifiers of genes for subnetwork with pattern [P=2]
# Create a data.frame from previous calculations (not shown)
# to allocate all relevant results:
metP2.ncon <- data.frame(
vi=c(2,1,3,6,4,27,5,7,8,26),
P2.id=c(11233,10543,15420,27031,16355,6967,27030,24773,33587,6072),
n.con=c(7,6,6,6,5,5,4,1,1,1),
n.TFcan=NA,
TF.can="",
stringsAsFactors=F)

# Note: meaning of metP2.ncon columns:
# "vi" - Vertex index (as shown in all figures)
# "P2.id" - Identifier of each one of the genes in the network
# "n.con" - Number of connections of that network
# "n.TFcan" - Number of TF candidates (to be filled)
# "TF.can" - Concatenated labels of TF candidates (to be filled)

# For that data.frame the following calculations were performed:
for(i in 1:10){
# Get the results of function g2g.TFcandidates
temp <- g2g.TFcandidates(tg.id=metP2.ncon$P2.id[i], min.fdr=0.1, min.r2=0.5, n.min.acc=12)
# Select only TF with positive value of correlation
temp <- temp$winn.summ[temp$winn.summ$r.pos==TRUE,]
# Order the data.frame by median of r in decreasing order
temp <- temp[order(temp$r.Med, decreasing=T),]
# Fill the variables needed:
# The number of TF candidates:
metP2.ncon$n.TFcan[i] <- nrow(temp)
# The label with all ids of the TFcandidates
metP2.ncon$TF.can[i] <- paste(temp$id, collapse=",")
}
```

```
> metP2.ncon[,1:4] # Show the first 4 columns
   vi P2.id n.con n.TFcan
1   2 11233     7      22
2   1 10543     6      19
3   3 15420     6      20
4   6 27031     6      19
5   4 16355     5      20
6  27  6967     5      19
7   5 27030     4      16
8   7 24773     1      24
9   8 33587     1      19
10 26  6072     1      27
# Variable TF.can contains TF candidates identifiers; see
> metP2.ncon$TF.can[1] # For the first case
[1] "20830,30554,25590,30563,10038,19302,21075,32973,16459,3529,28940,20355,18607,30900,
21120,31840,25181,525,1329,35383,27518,27181"

# Recover the TF candidates ids in a numeric vector
temp <- c() # An empty string
for(i in 1:10){
temp <- c(temp, as.integer(strsplit(metP2.ncon$TF.can[i], split=",", fixed=T)[[1]]))
}

# Now temp has all TF ids; let's see which ones are
# repeated and how many times:
> metP2tabTFcan <- table(temp)
> metP2tabTFcan <- metP2tabTFcan[order(metP2tabTFcan, decreasing=T)]

> length(metP2tabTFcan) # How many different TF?
[1] 43
> metP2tabTFcan # Ordered table
temp
 3529 10038 18607 20355 20830 21120 25590 30554 30563 30900 19302 21075
   10    10    10    10    10    10    10    10    10    10     9     9
25181   525 32973  9724  1329 25387 27120 27518 28940  8533 13495 22114
    9     8     8     6     5     4     4     4     4     3     3     3
 5799  6202  6269 27181 31840 33045 35383  1048  7175 11410 13256 13605
    2     2     2     2     2     2     2     1     1     1     1     1
16459 18840 24124 25818 27273 28948 33922
    1     1     1     1     1     1     1

# Note: we are going to consider ONLY TF candidates
# that are repeated 10 times, i.e., which are linked
# with all the 10 genes already in the subnetwork
> metP2bestTFids <- sort(as.integer(names(metP2tabTFcan[metP2tabTFcan==10])))
> metP2bestTFids
 [1]  3529 10038 18607 20355 20830 21120 25590 30554 30563 30900

 # metP2ids contains the identifiers of genes in pattern 2
> metP2ids
 [1] 10543 11233 15420 16355 27030 27031 24773 33587  6072  6967
```

```
# Obtains the core for the Gene2Gene algorithm
> P2wTF.core <- core.g2g(ids=c(metP2ids, metP2bestTFids))

# Filters results (with same parameters than before)
> P2wTF.g2g <- g2g(P2wTF.core, min.fdr=0.1, min.r2=0.7, m.a.quan=0.05, n.min.acc=12)

> analyze.g2g(P2wTF.g2g) # See results

Result of running function "g2g" with a set of 20 genes (ids in the input)
and with main parameters:
                              in.acc min.fdr min.r2 m.a.quan n.min.acc
1 AS,CM,CO,CQ,CW,JE,QC,QU,SR,ST,SY,ZU     0.1    0.7     0.05        12

There were 77 pairs of genes which surpassed the main parameters in at least 12 genotypes.
Those 77 pairs are formed by a total of 20 different genes.
Thus a total of 0 genes (0%) do not appear in the output.
The method estimated relations between 100% of the genes in the input.

Number of times that each gene id (upper row) participates into winner pairs (bottom row)
[Useful to detect "hub" genes].

Table of gene ids in winners.
10038 11233 27031  6967 10543 15420 27030 16355 20830 21120 25590 30554
   13    13    13     9     9     9     9     8     8     8     8     8
30563 24773 33587  3529  6072 18607 30900 20355
    8     7     6     4     4     4     4     2
Same table than above in rounded percentages:
Table of gene ids in winners.
10038 11233 27031  6967 10543 15420 27030 16355 20830 21120 25590 30554
    8     8     8     6     6     6     6     5     5     5     5     5
30563 24773 33587  3529  6072 18607 30900 20355
    5     5     4     3     3     3     3     1

Table of genotypes that are included in winners.
AS CM CO CQ CW JE QC QU SR ST SY ZU
77 77 77 77 77 77 77 77 77 77 77 77
Same table than above in rounded percentages:
Table of genotypes that are included in winners.
AS CM CO CQ CW JE QC QU SR ST SY ZU
 8  8  8  8  8  8  8  8  8  8  8  8

A total of 12 of the 12 genotypes (100%) are found in at least one winner.

Summary for the number of genotypes in each winner
   Min. 1st Qu.  Median    Mean 3rd Qu.    Max.
     12      12      12      12      12      12

Table of logical variable "r.pos"
TRUE
  77

Summary of average correlation coefficient by logical variable "r.pos".$`TRUE`
```

```
   Min. 1st Qu.  Median    Mean 3rd Qu.    Max.
 0.9446  0.9697  0.9790  0.9767  0.9865  0.9952


Summary of average Q values (for FDR) in all winners.
     Min.   1st Qu.    Median      Mean   3rd Qu.      Max.
2.809e-05 2.610e-04 5.617e-04 7.519e-04 1.044e-03 2.666e-03

Summary of average m.a (maximum absolute) by logical variable "r.pos".
$`TRUE`
   Min. 1st Qu.  Median    Mean 3rd Qu.    Max.
 0.1459  0.2476  0.3116  0.3203  0.3818  0.5654
-----------------------------------------------------------------------------------
```

References

Ashburner M, Ball CA, Blake JA, Botstein D, Butler H, Cherry JM, Davis AP, Dolinski K, Dwight SS, Eppig JT, et al. (2000) Gene ontology: tool for the unification of biology. *Nature genetics*, 25, 25–29.

Benjamini Y and Hochberg Y (1995) Controlling the false discovery rate: a practical and powerful approach to multiple testing. *Journal of the royal statistical society. Series B (Methodological)*, pp. 289–300.

Chen M and Thelen JJ (2011) Plastid uridine salvage activity is required for photoassimilate allocation and partitioning in arabidopsis. *The Plant Cell*, 23, 2991–3006.

Csardi G and Nepusz T (2006) The igraph software package for complex network research. *InterJournal, Complex Systems*, 1695, 1–9. URL `http://igraph.org`.

Escoto-Sandoval C, Flores-Díaz A, Reyes-Valdés MH, Ochoa-Alejo N, and Martínez O (2021a) A method to analyze time expression profiles demonstrated in a database of chili pepper fruit development. *Scientific Reports*, 11, 13181. doi:10.1038/s41598-021-92672-4.

Escoto-Sandoval C, Ochoa-Alejo N, and Martínez O (2021b) Inheritance of gene expression throughout fruit development in chili pepper. *Scientific Reports*, 11, 16. doi:https://www.nature.com/articles/s41598-021-02151-z.

Gómez-García MdR and Ochoa-Alejo N (2013) Biochemistry and molecular biology of carotenoid biosynthesis in chili peppers (capsicum spp.). *International journal of molecular sciences*, 14, 19025–19053.

Góngora-Castillo E, Ibarra-Laclette E, Trejo-Saavedra DL, and Rivera-Bustamante RF (2012) Transcriptome analysis of symptomatic and recovered leaves of geminivirus-infected pepper (capsicum annuum). *Virology journal*, 9, 1–16.

Hödl M and Basler K (2012) Transcription in the absence of histone h3. 2 and h3k4 methylation. *Current Biology*, 22, 2253–2257.

Jeevalatha A, Siddappa S, Kumar A, Kaundal P, Guleria A, Sharma S, Nagesh M, and Singh BP (2017) An insight into differentially regulated genes in resistant and susceptible genotypes of potato in response to tomato leaf curl new delhi virus-[potato] infection. *Virus research*, 232, 22–33.

Kwak KJ, Kim JY, Kim YO, and Kang H (2007) Characterization of transgenic arabidopsis plants overexpressing high mobility group b proteins under high salinity, drought or cold stress. *Plant and Cell Physiology*, 48, 221–231.

Lachmann A, Giorgi FM, Lopez G, and Califano A (2016) Aracne-ap: gene network reverse engineering through adaptive partitioning inference of mutual information. *Bioinformatics*, 32, 2233–2235.

Laurencelle L (2021) The exact binomial test between two independent proportions: A companion. *The Quantitative Methods for Psychology*, 17, 76–79.

Lee YRJ and Liu B (2004) Cytoskeletal motors in arabidopsis. sixty-one kinesins and seventeen myosins. *Plant Physiology*, 136, 3877–3883.

López-Kleine L, Leal L, and López C (2013) Biostatistical approaches for the reconstruction of gene co-expression networks based on transcriptomic data. *Briefings in functional genomics*, 12, 457–467.

Margolin AA, Nemenman I, Basso K, Wiggins C, Stolovitzky G, Dalla Favera R, and Califano A (2006) Aracne: an algorithm for the reconstruction of gene regulatory networks in a mammalian cellular context. In *BMC bioinformatics*, volume 7, pp. 1–15. BioMed Central.

Martinez O (2022) Time course gene expression experiments. In MA Ali and J Lee, editors, *Transcriptome Profiling: Progress and Prospects*, chapter 4, pp. 85–110. Elsevier.

Martínez O, Arce-Rodríguez ML, Hernández-Godínez F, Escoto-Sandoval C, Cervantes-Hernández F, Hayano-Kanashiro C, Ordaz-Ortiz JJ, Reyes-Valdés MH, Razo-Mendivil FG, Garcés-Claver A, and Ochoa-Alejo N (2021) Transcriptome analyses throughout chili pepper fruit development reveal novel insights into the domestication process. *Plants*, 10, 585.

Martínez O and Escoto-Sandoval C (2021) *Salsa: An R package of data mining facilities for* Capsicum *gene expression profiles.* doi:10.5281/zenodo.4767445. URL `https://zenodo.org/record/4767445#.Y81Lf-zMJjM`.

Ohler L, Niopek-Witz S, Mainguet SE, and Möhlmann T (2019) Pyrimidine salvage: Physiological functions and interaction with chloroplast biogenesis. *Plant physiology*, 180, 1816–1828.

Petricka JJ, Clay NK, and Nelson TM (2008) Vein patterning screens and the defectively organized tributaries mutants in arabidopsis thaliana. *The Plant Journal*, 56, 251–263.

R Core Team (2013) *R: A language and environment for statistical computing.* R Foundation for Statistical Computing, Vienna, Austria. URL `http://www.r-project.org`.

Redon C, Pilch D, Rogakou E, Sedelnikova O, Newrock K, and Bonner W (2002) Histone h2a variants h2ax and h2az. *Current opinion in genetics & development*, 12, 162–169.

Reverter A and Chan EK (2008) Combining partial correlation and an information theory approach to the reversed engineering of gene co-expression networks. *Bioinformatics*, 24, 2491–2497.

Rhee SY, Wood V, Dolinski K, and Draghici S (2008) Use and misuse of the gene ontology annotations. *Nature Reviews Genetics*, 9, 509.

Song Q, Chen S, Wu Y, He Y, Feng J, Yang Z, Lin W, Zheng G, Li Y, and Chen H (2020) Comparative transcriptome analyses of gene response to different light conditions of camellia oleifera leaf using illumina and single-molecule real-time-based rna-sequencing. *Forests*, 11, 91.

Spies D and Ciaudo C (2015) Dynamics in transcriptomics: advancements in rna-seq time course and downstream analysis. *Computational and structural biotechnology journal*, 13, 469–477.

Spies D, Renz PF, Beyer TA, and Ciaudo C (2019) Comparative analysis of differential gene expression tools for rna sequencing time course data. *Briefings in bioinformatics*, 20, 288–298.

Strober B, Elorbany R, Rhodes K, Krishnan N, Tayeb K, Battle A, and Gilad Y (2019) Dynamic genetic regulation of gene expression during cellular differentiation. *Science*, 364, 1287–1290.

Wang N, Yang Y, Moore MJ, Brockington SF, Walker JF, Brown JW, Liang B, Feng T, Edwards C, Mikenas J, et al. (2019) Evolution of portulacineae marked by gene tree conflict and gene family expansion associated with adaptation to harsh environments. *Molecular biology and evolution*, 36, 112–126.

Zhang Y, Li Z, Ma B, Hou Q, and Wan X (2020) Phylogeny and functions of lob domain proteins in plants. *International Journal of Molecular Sciences*, 21, 2278.

Zhang Z, Liu J, Huber DJ, Qu H, Yun Z, Li T, and Jiang Y (2021) Transcriptome, degradome and physiological analysis provide new insights into the mechanism of inhibition of litchi fruit senescence by melatonin. *Plant Science*, 308, 110926.

Zhao X, Bai X, Jiang C, and Li Z (2019) Phosphoproteomic analysis of two contrasting maize inbred lines provides insights into the mechanism of salt-stress tolerance. *International journal of molecular sciences*, 20, 1886.

Zoppoli P, Morganella S, and Ceccarelli M (2010) Timedelay-aracne: Reverse engineering of gene networks from time-course data by an information theoretic approach. *BMC bioinformatics*, 11, 1–15.
